# hemaClass.org: Online One-By-One Microarray Normalization and Classification of Hematological Cancers for Precision Medicine

**DOI:** 10.1371/journal.pone.0163711

**Published:** 2016-10-04

**Authors:** Steffen Falgreen, Anders Ellern Bilgrau, Rasmus Froberg Brøndum, Lasse Hjort Jakobsen, Jonas Have, Kasper Lindblad Nielsen, Tarec Christoffer El-Galaly, Julie Støve Bødker, Alexander Schmitz, Ken H. Young, Hans Erik Johnsen, Karen Dybkær, Martin Bøgsted

**Affiliations:** 1 Department of Haematology, Aalborg University Hospital, Aalborg, Denmark; 2 Department of Clinical Medicine, Aalborg University, Aalborg, Denmark; 3 Department of Hematopathology, University of Texas MD Anderson Cancer Center, Houston, United States of America; Cornell University, UNITED STATES

## Abstract

**Background:**

Dozens of omics based cancer classification systems have been introduced with prognostic, diagnostic, and predictive capabilities. However, they often employ complex algorithms and are only applicable on whole cohorts of patients, making them difficult to apply in a personalized clinical setting.

**Results:**

This prompted us to create hemaClass.org, an online web application providing an easy interface to one-by-one RMA normalization of microarrays and subsequent risk classifications of diffuse large B-cell lymphoma (DLBCL) into cell-of-origin and chemotherapeutic sensitivity classes. Classification results for one-by-one array pre-processing with and without a laboratory specific RMA reference dataset were compared to cohort based classifiers in 4 publicly available datasets. Classifications showed high agreement between one-by-one and whole cohort pre-processsed data when a laboratory specific reference set was supplied. The website is essentially the R-package **hemaClass** accompanied by a Shiny web application. The well-documented package can be used to run the website locally or to use the developed methods programmatically.

**Conclusions:**

The website and R-package is relevant for biological and clinical lymphoma researchers using affymetrix U-133 Plus 2 arrays, as it provides reliable and swift methods for calculation of disease subclasses. The proposed one-by-one pre-processing method is relevant for all researchers using microarrays.

## Introduction

In addition to current clinically used risk factor scoring systems, several independent gene expression profile (GEP) based risk stratifications have been proposed, with biological and clinical significance in hematological cancers. Although drug targetable genes, which are only expressed in subtypes of e.g. DLBCL tumours have been identified, they are not readily applicable in clinical research and routine settings due to a lack of available routine diagnostic tests [1, 2].

[3] developed an important example of a biological sub-classification of lymphoma. On the basis of GEP analyses, DLBCL cases were classified as activated B-cell phenotype (ABC) or germinal center B-cell phenotype (GCB) with different clinical outcomes. The validity of this classification and its prognostic importance have been confirmed in a number of later studies [4–8]. Recently, we have refined the ABC/GCB subclassification of DLBCL to include a B-cell Associated Gene Signature (BAGS) classifier capable of classifying DLBCL samples into 5 different B-cell subtypes: Naive (N), Centrocyte (CC), Centroblast (CB), Memory (M), and Plasmablasts (PB) [9]. The BAGS classifier stratifies the GCB phenotype into CC and CB subtypes, with superior survival in the CC subtype. Thus, different treatment regimes could be considered in subsets of the GCB class of patients. In another study we developed classification based resistance gene signatures (REGS) for the most prominent drugs used in the treatment of DLBCL patients: Cyclophosphamide (C), Doxorubicin (H), and Vincristine (O) [10]. However, these and most existing algorithms are only applicable in the presence of whole cohorts of patients, making them difficult to apply in a routine clinical setting.

The traditional lymphoma staging and risk classification systems are based on the Ann Arbor classification for staging of lymphoma (extent of disease and extranodal involvement) and simple prognostic tools such as the international prognostic index (IPI, [11]) for large cell lymphoma and the Follicular Lymphoma International Prognostic Index (FLIPI, [12]), both derived from patient age, performance status, easy available blood tests, and disease stage. Due to the simplicity of these clinical risk stratification algorithms they are still the most widely used risk scoring systems today. Risk stratification according to these algorithms has been systematized and made easily accessible for desktop, online, and even smart-phone use. Easily accessible molecular classification methods are, however, lagging behind, thereby delaying the translation of new molecular findings into clinical practice. A few methods exist for cancer types other than lymphoma, including acute myeloid leukemia (AML) [13], and for lymphoma [14] has developed an ABC/GCB classifier, which is stable across microarray technologies and trial centres. This ABC/GCB classifier is, however, potentially biased towards classes which differentiate the prognosis instead of biological classes, since the ensemble of classifiers were chosen based on their ability to separate survival.

In clinical settings, the methods need to be applicable for a single patient sample and straightforward to use. This prompted us to develop a user-friendly and flexible web-based tool for ABC/GCB, BAGS, and REGS classification using our recently developed classifiers for microarray data based on the Affymetrix’s Human Genome U-133 Plus 2 array. The classifications made by the web-based tool hemaClass.org are compared to the existing state-of-the-art and approved ABC/GCB classifications of DLBCL. We believe that hemaClass.org will provide a novel and user-friendly concept for bringing complex molecular classification of diseases more swiftly into daily clinical practice.

## Methods

### Classification workflow

**Fig 1:**
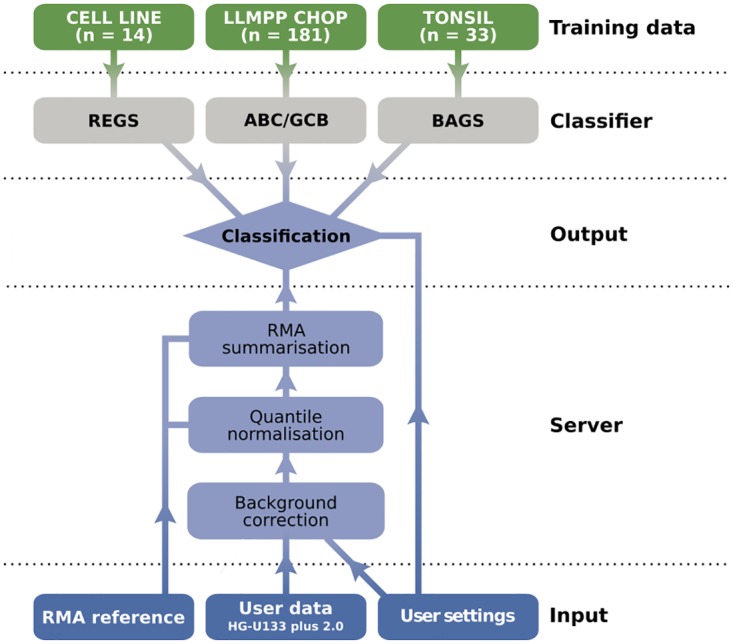
Diagram of the hemaClass.org workflow architecture.

The workflow architecture of hemaClass.org is illustrated in Fig 1. The user is presented with a graphical interface for uploading data and adjusting settings. The user data is RMA pre-processed by the server (with an optional user one-by-one RMA reference), and subsequently processed by the classification algorithms. The results are then returned for download and inspection via the user interface.

### Software availability and technical details

The interactive web application available at hemaclass.org was created using the statistical programming language R [15], the software package **shiny** [16], and the accompanying Linux server software. All hemaClass.org functionality, including the RMA normalization and classification procedures, are available through the accompanying package **hemaClass** based on a number of packages from the Comprehensive R Archive Network [15] and the Bioconductor environment [17]. The Shiny server handles the interaction between the front end web application and the back end R processing. The back end is essentially the well-documented **hemaClass** package which can be utilized as a programmatical interface to the functionality of the website. However, the package also allows users to run a local instance of the website if one wishes to avoid uploading large files to our server. The development and latest version of **hemaClass** is open source and freely available at https://github.com/oncoclass/hemaclass for sharing, modification, and redistribution. All bug-reports, suggestions, and comments on the website or package are welcome and should be posted to the github page following the link above. The regular RMA pre-processing is carried out with the Bioconductor package **affy** [18]. Core functions for the one-by-one RMA pre-processing are written in C++ and imported to R using **Rcpp** and **RcppArmadillo** [19–22].

### Data overview

The seven gene expression datasets used in this paper are summarized in Table 1. All GEP data are from the Affymetrix GeneChip HG-U133 Plus 2.0 array and available at the Gene Expression Omnibus (GEO) [23] website (http://www.ncbi.nlm.nih.gov/geo/). To establish the classifiers the following datasets are used:

1 Gene expressions from 181 CHOP treated DLBCL patients are used to establish the ABC/GCB classifier. This cohort will be referred to as the *LLMPP CHOP* (Lymphoma/Leukemia Molecular Profiling Project CHOP) cohort [7]. The cohort is also used as a default reference set throughout the paper for one-by-one RMA normalization of arrays.
2 The BAGS classifier is based on gene expression data from eight human tonsils sorted in five B-cell subsets. This dataset is also used for scaling of gene expression data for BAGS classification, and will be referred to as the *Tonsil dataset* [9].
3 The REGS classifiers are based on a panel of 12 Multiple Myeloma (MM) and 14 DLBCL cell lines. This panel will be referred to as *BCELL26*. The DLBCL part of the cell line panel is used for scaling of patient data and will be referred to as *DLBCL14* [10].

For validation the following four DLBCL cohorts are used:

4 The Aalborg OCT cohort (*CHEPRETRO*) of 89 Danish DLBCL patients undergoing first-line treatment at Aalborg University Hospital [9].
5 The International DLBCL Rituximab-CHOP Consortium MD Anderson (*IDRC*) cohort of 470 DLBCL patients treated with R-CHOP first-line therapy [24]. Note, that these samples are formalin-fixed, paraffin-embedded (FFPE).
6 The Lymphoma/Leukemia Molecular Profiling Project R-CHOP (*LLMPP R-CHOP*) cohort of 233 DLBCL patients treated with R-CHOP first-line therapy [7].
7 The Mayo-Dana-Farber Cancer Institute (*MDFCI*) cohort of 90 DLBCL patients treated with R-CHOP first-line therapy [8].

The GEO datasets were downloaded using the R-package **DLBCLdata** [25].

**Table 1:** Overview of used datasets and GEO accession numbers.

No.	Dataset	*n*	Usage	GEO number	Ref.
1.	LLMPP CHOP	181	Training	GSE10846	[7]
2.	Tonsil	33	Training	GSE56315	[9]
3.	BCELL26	26	Training	GSE53798	[10]
4.	CHEPRETRO	89	Validation	GSE56315	[9]
5.	IDRC	470	Validation	GSE31312	[24]
6.	LLMPP R-CHOP	233	Validation	GSE10846	[7]
7.	MDFCI	90	Validation	GSE34171	[8]

### One-by-one RMA normalization

Robust multichip average (RMA) pre-processing consists of three steps in the order: (1) Background correction, (2) quantile normalization, and (3) summarization of probes to probe-sets [26, 27]. Confer [28] for a comprehensive account on RMA. Both the quantile normalization and summarization procedures of RMA are cohort based and hence need to be altered to facilitate a one-by-one RMA pre-processing scheme. Previous approaches similar to the one-by-one normalization approach used by hemaClass.org have been described by [29] and [30] As quantile normalizer, the empirical cumulative distribution function (ECDF) of the mean of the sample quantiles of an RMA background corrected reference dataset is used in place of the usually applied ECDF of the mean of the sample quantiles of the user supplied data [31]. To mimic the summarization procedure of RMA [27] the probe effects estimated by median polish for the same reference data is subtracted all probes of the user data. The RMA pre-processed expression value for each probe-set is then estimated as the median of the associated probes. For more detail on our one-by-one normalization approach see S1 Text section S4. Finally, before classification the median of each probe-set in the RMA reference dataset is subtracted from the corresponding probe-set in the user data, since the classifiers were trained on median centered data.

The one-by-one normalization has the implicit assumption that the samples and reference follow the same distribution. This assumption might be violated by batch effects arising from differences in laboratory specific sample preparations and can cause severe bias in the normalization; to accomodate this hemaClass.org allows users to upload their own RMA reference dataset prepared under similar conditions. In the this paper a laboratory specific RMA reference is referred to as an InLab reference. InLab references were simulated by selecting a random subset of 30 samples from each cohort. Samples were also one-by-one RMA normalized using the LLMPP CHOP dataset as an external reference; this is referred to as ExLab reference normalization.

### Classification methods

#### Elastic nets

Logistic and multinomial regression were used in all classification methods available at hemaClass.org. However, in GEP experiments, the number of probe-sets present on the microarray always outnumbers the sample size. Collinearity present among the features further aggravates the problem of identifying genes responsible for the underlying biological mechanism. Regression under these ill-posed circumstances is typically handled by so-called regularization. Here the elastic net penalty [32, 33], which is a combination of the Lasso [34] and ridge regression [35], was used. Similar to the Lasso, this penalty ensures simultaneous variable selection and model estimation by forcing small coefficients to be zero, yielding sparse solutions, but contrary to the Lasso the elastic net penalty is capable of selecting more variables than samples.

The elastic net penalty contains two parameters *α* and *λ*. The parameter *α* interpolates the elastic net penalty between the ridge and the Lasso penalty which corresponds to values of 0 and 1, respectively. The parameter *λ* determines the amount of shrinkage of the coefficients with larger values inducing more shrinkage until no variables are contained in the model. Regularized logistic and multinomial regression were performed with the R-package **glmnet** [32].

#### ABC/GCB classification

The ABC/GCB classifier was established using logistic regression with an elastic net penalty on the LLMPP CHOP cohort. Of the 181 patients 74 were ABC, 76 were GCB, and 31 were non-classified. Using the 150 patients classified as either ABC or GCB, a dichotomous classifier capable of assigning each sample an estimate of the probability of being ABC was established.

To avoid over-fitting and limit the number of noise contributing genes, the elastic net parameters *α* and *λ* were chosen through 10 fold cross-validation. The parameter *α* was varied between 0.1 and 1 with step size 0.025 and log(*λ*) was varied between −10 and 2 with step size 0.06. The optimal combination of the parameters, and thereby the number of probe-sets, was found at the values minimizing the deviance. The results of the cross validations are shown in S1 Fig. The minimum deviance of 0.13 was attained at *α* = 0.15 and log(*λ*) = −7.29. This resulted in a gene expression classifier consisting of 381 probe-sets corresponding to 273 Ensembl Gene IDs.

When a tumour sample was classified according to the ABC/GCB classifier using cohort or InLab one-by-one normalized data the associated gene expressions are rescaled probe-set wise by the standard deviation of the LLMPP CHOP data divided by the standard deviation of the cohort or InLab reference data. For ExLab one-by-one normalization the data was used directly, since the training data for the ABC/GCB classifier was the same as the ExLab reference in the current study.

#### Wright’s ABC/GCB Classification

Standard GEP ABC/GCB classification is done using Wright’s naive Bayes classifier. This method is not included on hemaClass.org, but is used in the current study for comparison of results from our elastic net classifier.

Bayesian compound covariate classification [36] with probeset list, weights and prior probabilities as described by [7] was used to perform ABC/GCB classification (specific details obtained by personal communication with George Wright). In addition to this the probesets were brought to the same scale as Lenz et al.’s [7] probesets by a rescaling of the probeset-wise standard deviation.

#### REGS classification

In the paper by [10] REGS classifiers were established for prediction of resistance to the drugs C, H, and O. The classifiers were established on BCELL26 using regularized logistic regression analogous to the procedure described for the aforementioned ABC/GCB classifier. The number of microarray probes and corresponding genes for each of the REGS classifiers is shown in S5 Table.

The probability of resistance to the combination therapy, *p*_*CHO*_, was estimated based on the probabilities of drug resistance toward each of the three drugs: *P*_*C*_, *P*_*H*_, and *P*_*O*_, respectively. This probability is calculated as the posterior probability of being resistant, given resistance towards each of the individual drugs under the assumption of conditional independence and uniform priors. The formula is also known as Graham’s formula:

$$\begin{array}{c} P_{CHO}=\frac{P_{C}P_{H}P_{O}}{P_{C}P_{H}P_{O}+\left(1-P_{C}\right)\left(1-P_{H}\right)\left(1-P_{O}\right)}. \end{array}$$

Derivation of the formula is shown in S1 Text section S2. If a drug is left out in the combination therapy the drug is simply removed from the formula. This appoach to resistance to the combination therapy was used in [10].

When a tumour sample is classified according to the REGS classifiers the associated gene expressions are rescaled probe-set wise by the standard deviation of DLBCL14 divided by the standard deviation of the cohort, InLab, or ExLab RMA reference dataset.

Resistance classifiers for other chemotherapeutic drugs and diseases are also available on hemaClass.org, though established elsewhere [37–39]. The Rituximab sensitivity classifier of [39] and [40] uses an elastic net approach, as above, but with three classes. The Melphalan sensitivity classifier of [37] uses sparse partial least squares to classify samples as either “sensitive”, “intermediate” or “resistant”. This classifier was developed for multiple myeloma (MM) patients and was thus based on other data [37].

#### BAGS classification

The BAGS classifier established by [9] was based on multinomial regression regularized by an elastic net penalty. The classifier was trained on the Tonsil dataset in a manner similar to the ABC/GCB classifier. The BAGS classifier uses 327 probes corresponding to 205 Ensembl Gene IDs.

When a tumour sample is classified according to the BAGS classifier the associated gene expressions are rescaled probe-set wise by the standard deviation of the Tonsil data divided by the standard deviation of the cohort, InLab, or ExLab one-by-one reference dataset. The rescaling is performed to make the data comparable to the Tonsil dataset.

### Inter-method reproducibility assessments

To evaluate the reproducibility of the class probabilities obtained through cohort or reference based RMA normalization, Pearson’s correlation coefficient for the logit-transformed probabilities and 95% confidence interval (CI) were calculated for each classifier and dataset. The identity and *total* least square regression lines were compared to assess bias in the estimated probabilities [41]. Total least squares regression was used as errors are present in both classification probabilities.

For each classifier the associated categories were obtained by thresholding the estimated probabilities. The ABC/GCB classifier was thresholded by 0.1 and 0.9, i.e. a tumour sample was classified as ABC when the estimated probability exceeded 0.9, GCB when it was below 0.1, and unclassified otherwise. For the BAGS classifier a tumour was classified as the class N, CB, CC, M, or PB with the highest probablity, if the associated probability exceeded 0.5 and unclassified when this threshold was not met for any subtype. For the REGS classifiers, C, H, O, and CHO combined, the thresholds were the 33% and 66% percentile of the estimated probabilities. The classifiers were applied to datasets using cohort, InLab, and ExLab one-by-one RMA normalization. Confusion matrices tabulating classifications from cohort normalized data versus InLab or ExLab normalized data were created, and from these the Accuracy (percent with similar classification to cohort), Cohen’s weighted *κ*, and corresponding 95% CIs were computed to assess the agreement between the determined classes.

## Results

### Using hemaClass.org

The website is an easy-to-use, interactive interface for the **hemaClass** package with the desired RMA normalization and the classification methods selected by the user. The usage of the website is largely self-explanatory with context-dependent boxes aiding users with further information, warnings, or errors. A comprehensive tutorial and guide to both the website and package is provided on the website or by running vignette(“howto”) in R. Uploaded patient samples are normalized and classified depending on settings chosen by the user.

### ABC/GCB classification

In order to classify patients as ABC/GCB based on the implemented one-by-one normalization method a classifier based on the regularised logistic regression was established. The classifications were evaluated in the four clinical cohorts CHEPRETRO, MDFCI, IDRC, and LLMPP R-CHOP, which have all been classified according to Wright’s naive Bayes classifier [7, 9, 36]. The rates of agreement between the two classifiers based on cohort normalized data are shown in Table 2. Note that unclassified samples were included in the estimation of this rate i.e. a patient classified as ABC by one classifier but unclassified by the other is considered an error. The table also includes the alternative measure of agreement, Cohen’s weighted *κ*, where misclassifications involving the unclassified group are weighted by 1/2. High agreement between the two classifiers are observed for CHEPRETRO and LLMPP R-CHOP, while the accuracy and Cohen’s weighted *κ* are lower for IDRC and MDFCI. The accompanying confusion matrices are shown in the first rows of S1 Table.

**Table 2:** Comparison of ABC/GCB classification performed using Wright’s naive Bayes classifier [36] and the established elastic net classifier both based on cohort normalization. The second column shows the accuracy of the classifiers with 95% CI. The third column shows the Cohen’s weighted *κ* with 95% CI.

Dataset	Accuracy	Cohen’s *κ*
CHEPRETRO	0.94 (0.87, 0.98)	0.94 (0.88, 1.00)
MDFCI	0.78 (0.68, 0.86)	0.77 (0.66, 0.88)
IDRC	0.82 (0.79, 0.86)	0.80 (0.76, 0.85)
LLMPP R-CHOP	0.91 (0.86, 0.94)	0.90 (0.85, 0.96)

**Fig 2:**
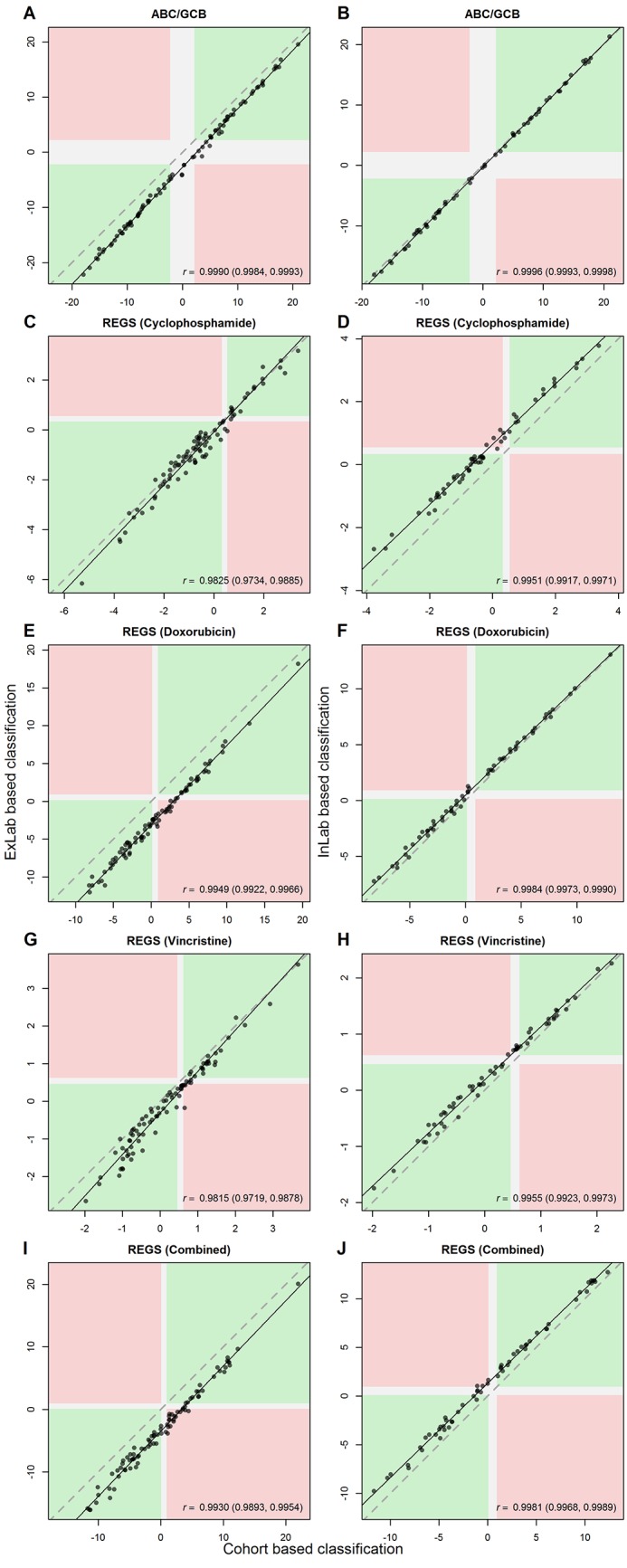
Comparison of logit probabilities for the ABC/GCB and REGS classifiers obtained through InLab or ExLab one-by-one normalization against cohort normalization. The areas marked with green indicate patients with similar classification between cohort based normalization and ExLab one-by-one normalization (A, C, E, G, I), or InLab one-by-one normalization (B, D, F, H, J). The areas marked with red indicate complete misclassifications. For ABC/GCB and REGS the white areas indicate unclassified and intermediate sensitivity, respectively, in at least one of the classifiers. The dashed and solid line show the identity and total least squares line, respectively.

The logit probability of being ABC estimated using the established cohort-based classifier was compared to the corresponding estimate obtained through the ExLab one-by-one normalized classification scheme in Fig 2A for CHEPRETRO. The probabilities estimated through the two methods are very similar, but values from ExLab normalization are slightly uncalibrated (or biased) and skewed downwards, indicating that different cut points might be used for the classifications. For InLab one-by-one normalization this error and bias is greatly minimized as shown in Fig 2B.

For both methods, patients are classified as ABC when the estimated probability exceeds 0.9 and GCB when it is below 0.1. In Table 3 the resulting classifications for the four validation datasets are compared in terms of accuracy and Cohen’s weighted *κ* for cohort, using either Wright’s Bayes classifier or the elastic net classifier, against InLab or ExLab RMA reference normalization. For ExLab normalization CHEPRETRO and LLMPP R-CHOP both show a high Cohen’s weighted *κ* and accuracy considering that misclassifications involving unclassified samples count as errors, while values are moderate for MDFCI and IDRC when comparing to cohort based classifications for both the elastic net and Wright’s classifier. The reduced rate of agreement and Cohen’s weighted *κ* using ExLab one-by-one normalization in IDRC may be due to the samples being FFPE although this seems to be remedied by InLab one-by-one normalization. Accuracy and Cohen’s weighted *κ* are very high when comparing InLab based classifications to cohort based classifications for the elastic net classifier, but values are still moderate for MDFCI when comparing against Wright classifications. The accompanying confusion matrices for the elastic net classifier are shown in the lower part of S1 Table. Note that changes in predicted classes are mainly due to shifts into NC from ABC or GCB. Direct disagreements between the classifiers are seemingly rare and only occurs in the IDRC dataset.

**Table 3:** Comparison of classifications obtained using cohort based normalization against Exlab and InLab reference based normalization. The classifications are compared in terms of accuracy, Cohen’s weighted *κ*, and Pearson’s correlation coefficient *r* all supplied with 95% CIs. The comparisons in the first and last three columns are based on the ExLab and InLab reference based normalization method, respectively. For ABC/GCB classification, results from InLab or Exlab classification with the elasitic net classifier is compared against ABC/GCB classes for cohort normalized data obtained using both Wrights Bayes classifier and the elastic net classifier.

	ExLab RMA pre-processing			InLab RMA pre-processing		
	Accuracy	Cohen’s *κ*	Pearson’s *r*	Accuracy	Cohen’s *κ*	Pearson’s *r*
**ABC/GCB (Wright)**						
CHEPRETRO	.89 (.80, .94)	.89 (.79, .98)	-	.97 (.88, 1.)	.97 (.90, 1.)	-
MDFCI	.63 (.52, .73)	.52 (.40, .64)	-	.72 (.59, .83)	.71 (.55, .86)	-
IDRC	.67 (.63, .71)	.62 (.56, .67)	-	.84 (.80, .87)	.82 (.77, .86)	-
LLMPP R-CHOP	.83 (.77, .87)	.82 (.74, .89)	-	.88 (.83, .92)	.88 (.82, .93)	-
**ABC/GCB**						
CHEPRETRO	.88 (.79, .94)	.87 (.78, .97)	.999 (.998, .999)	.98 (.91, 1.)	.98 (.93, 1.)	1. (.999, 1.)
MDFCI	.69 (.59, .78)	.68 (.53, .82)	.998 (.998, .999)	.98 (.91, 1.)	.98 (.85, 1.)	1. (.999, 1.)
IDRC	.65 (.61, .69)	.62 (.57, .68)	.986 (.983, .988)	.93 (.91, .95)	.93 (.90, .96)	.993 (.991, .994)
LLMPP R-CHOP	.82 (.77, .87)	.82 (.74, .89)	.999 (.999, .999)	.94 (.90, .97)	.94 (.90, .98)	.991 (.988, .993)
**BAGS**						
CHEPRETRO	.58 (.47, .69)	.56 (.28, .84)	-	.78 (.65, .88)	.74 (.33, 1.)	-
MDFCI	.54 (.43, .64)	.48 (.17, .79)	-	.80 (.68, .89)	.83 (.30, 1.)	-
IDRC	.52 (.47, .56)	.41 (.32, .50)	-	.79 (.75, .83)	.79 (.62, .96)	-
LLMPP R-CHOP	.56 (.49, .62)	.53 (.36, .70)	-	.88 (.82, .92)	.88 (.60, 1.)	-
**REGS**						
CHEPRETRO	.73 (.68, .78)	.71 (.64, .77)	.934 (.920, .946)	.84 (.79, .88)	.83 (.76, .89)	.992 (.990, .994)
MDFCI	.60 (.55, .65)	.55 (.48, .61)	.824 (.788, .855)	.90 (.86, .94)	.89 (.83, .96)	.997 (.996, .997)
IDRC	.52 (.49, .54)	.33 (.30, .36)	.660 (.635, .685)	.85 (.84, .87)	.84 (.81, .86)	.981 (.979, .983)
LLMPP R-CHOP	.58 (.54, .61)	.50 (.46, .54)	.810 (.786, .831)	.90 (.87, .92)	.89 (.85, .92)	.992 (.990, .993)

### REGS classification

The probability of sensitivity towards each of the three drugs C, H, and O was estimated using hemaClass.org for both InLab and ExLab one-by-one normalization. The logit probabilities of sensitivity are plotted against those obtained by cohort based normalization in Fig 2 for CHEPRETRO data. Panels C, E, G, and I show the plots based on ExLab normalization and panels D, F, H, and J show the plots for the InLab based normalization. The probabilities obtained by ExLab and cohort based normalization are comparable, but similar to the other classifiers ExLab normalization leads to slightly skewed and biased probabilities, indicating that different cut points should be considered. The probabilities obtained by the InLab one-by-one normalization resembles the cohort based to a great extent, indicating that similar well-calibrated probabilities are obtainable for different laboratories by supplying an InLab reference set.

Based on the estimated probabilities, the patients were categorised as sensitive, intermediate, or resistant based on the thresholds specified in Section. The classes obtained by hemaClass.org are compared to those obtained by the cohort based approach in Table 3 in terms of rate of agreement and Cohen’s weighted *κ*. Low to moderate rates of agreement are observed for the ExLab normalized data, and again the InLab one-by-one approach yielded higher agreement with classifications obtained from cohort based normalization. The associated confusion matrices for InLab and ExLab one-by-one normalization are shown in S3 and S4 Tables, respectively.

### BAGS classification

**Fig 3:**
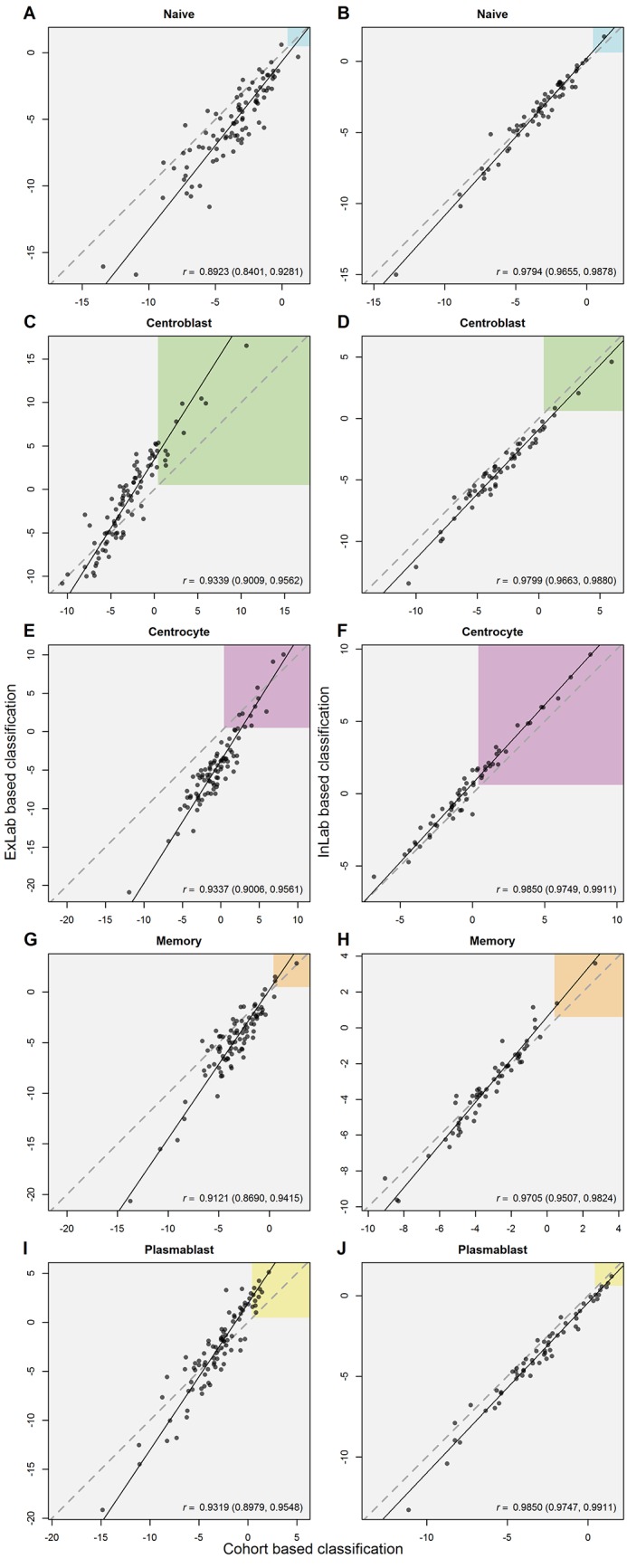
Comparison of logit probabilities for the BAGS classifier obtained through ExLab or InLab one-by-one normalization against cohort normalization. The coloured regions in the figure correspond to a threshold probability of 0.5. The dashed and solid line show the identity and total least squares line, respectively.

The BAGS classifier was evaluated in a manner similar to the ABC/GCB classifier. The logit probability of a patient’s tumour originating from one of the five subpopulations was estimated by means of cohort, InLab, and ExLab one-by-one normalization. The logit probabilities estimated by the ExLab normalization are plotted against logit probabilities from cohort based normalization in Fig 3 panels A, C, E, G, and I for CHEPRETRO. The correlations between the logit probabilities are highly significant, but also skewed and biased. The logit probabilities estimated using InLab one-by-one normalization are plotted against logit probabilities for the cohort based normalization in Fig 3 panels B, D, F, H, and J for CHEPRETRO. The InLab one-by-one normalization removes much of the aforementioned bias.

Based on the probabilities estimated by means of each of the three normalization methods the patients of the four clinical cohorts are grouped into the BAGS. The rates of agreement between the cohort based, and InLab or ExLab based classifications are shown in Table 3 along with Cohen’s weighted *κ*. For the BAGS classifier low rates of agreement were also found when comparing results from ExLab normalization with cohort normalization, while InLab normalization again led to improved agreement. The associated confusion matrices are shown in S2 Table.

## Discussion

Despite the enormous amount of resources spent on developing molecular based cancer classification systems, most of these are still not available in daily clinical practice. To allow for fast validation of our recent findings [9, 10], we have developed an easily accessible web application that permits other users to apply ABC/GCB, BAGS, and drug resistance classification to their own datasets. Since GEP classifiers rely on RMA normalized data, we also implemented a reference based RMA normalization that allows samples to be pre-processed one-by-one instead of in entire cohorts.

One-by-one normalization was done using both an external RMA reference (ExLab) and a mimicked laboratory specific reference (InLab). Classifications obtained through one-by-one pre-processing performed by hemaClass.org were then compared to those obtained using cohort based normalization in four clinical cohorts. The results showed that a one-by-one array analysis approach is feasible and performs comparably with the whole cohort based method when an InLab reference is used, while differences between ExLab and cohort normalized classifications were too large to be satisfactory. Users are thus encouraged to supply their own reference for RMA pre-processing. It seems that this approach allows for a realistic application of microarray based lymphoma classification for research projects, and after suitable standardisation and calibration, even for clinical use.

The poorer results for ExLab one-by-one normalization and classification is likely caused by not accounting for lab-specific batch effects in the normalization process. To check for incorrect normalization hemaClass.org calculates the inter-quartile range (IQR) of the relative log expression (RLE) [42] across probe-sets of one-by-one normalized arrays. Large values of the RLE IQR indicates low-quality arrays resulting from e.g. incorrect laboratory procedures for cohort normalized data, but can also indicate normalization against an improper reference for one-by-one normalized data, and can thus be used in cases with uncertainty of the validity of a user supplied reference. Based on the validation in S1 Text section S6 we recommend removing samples from the analysis if the RLE IQR exceeds a value of 0.6. Users are, however, still encouraged to take proper precautions when selecting an RMA reference. For labs using a fixed InLab reference, the RLE IQR could also be used as a means of statistical process control. If RLE IQR values start to increase over time, this could indicate changes in laboratory procedures that might have to be adressed.

The present treatment algorithms for DLBCL are based on disease stage and clinical risk stratification without accounting for the underlying tumour-biology [43] and does not routinely account for the enormous variations in tumor biology between patients. The CHOP combination therapy (cyclophosphamide, doxorubicin, vincristine, and prednisone) has been the backbone of DLBCL therapy for decades with the only significant improvement being the addition of monoclonal CD20 antibodies (Rituximab) [44]. Despite the addition of antibody therapy to conventional chemotherapy only 55% of patients with poor risk disease achieve durable remission [45]. Thus, the need for new therapeutic options in DLBCL is obvious. Currently a number of new drugs have shown promising activity in DLBCL, but their role outside clinical trials have not been defined. These drugs are different from conventional chemotherapeutic compounds by targeting specific deregulated cell-cycle pathways [46]. An important example is inhibition of the NF-*κ*B pathway by proteasome inhibitors (i.e. bortezomib). Interestingly, the constitutive activation of the NF-*κ*B pathway is characteristic for the ABC subtype of DLBCL which consequently enhances the effect of bortezomib in this subtype [47].

With the increasing number of new drugs likely to become available over the next years and the fact that their efficacy may vary between subsets of patients defined by gene expression profiles, the current treatment of patients based on disease stage and clinical information alone will not be sufficient. hemaClass.org provides an example of fast processing of complex molecular information in a way that is simple and readily at hand for clinicians.

From a practical perspective a couple of challenges have to be addressed. First, the prognostic potential of the classifiers in these types of analyses has been established on specific tissues, which means one has to trust that the right tissue has been extracted and handled correctly through all the steps in the laboratory ending up with a reference array data set of sufficient quality. Our reference data have for instance been controlled by looking at the frequency of ABC/GCBs and BAGS classes and their survival curves as well as tissue control by experienced pathologists. Second, one has to address the need for a reference dataset for one-by-one RMA pre-processing established under similar conditions as the samples one wishes to classify. However, calibration of laboratory equipment is a well-known issue for many experimental techniques used in molecular biology like qPCR, mass spectrometry, immunohistochemistry, and flow cytometry. An important part of the calibration is that samples should be calibrated towards a dataset consisting of a representative set of tissue samples. We suggest that these challenges could be addressed by establishing a central tissue bank with officially approved data, e.g. by an international medical consortium, for the specific disease, similar to The Gene Expression Barcode [48] where consensus data for many tissues can already be found.

Alternatively the frozen RMA (fRMA) approach suggested by [30] could be used. This approach allows samples to be RMA normalized one-by-one against a frozen reference established across many different tissues and laboratories, taking the variation across laboratories and tissues for single probes into account. The current implementation of fRMA does not center and scale the data, so it cannot be used with the classifiers implemented in hemaClass.org, but had the training data for the classifiers been normalized with fRMA this might eliminate the need for centering and scaling sample data.

Another limitation of the current web application is that hemaClass.org only works with Affymetrix HG-U133 Plus 2.0 microarrays. This can, however, be circumvented by either re-annotation to HUGO Gene Nomenclature Committee (HGNC) approved symbols as suggested by [14] or by re-annotated chip definition files as suggested by [49]. At the moment we are working on extending the web application to other array types along these lines. In the future, gene expressions will likely be measured using RNA-seq technology instead of microarrays. By summarizing the expression levels at gene-annotations rather than affymetrix probes and scaling the data it might also be possible to use microarray based classifiers with RNA-seq data. Alternatively the transcriptome, for the training data used for establishing the BAGS and REGS classifiers, would have to be measured with RNA-seq and the classifiers retrained.

Traditionally ABC/GCB classification has been achieved using the naive Bayes classifier of [36] which is based on cohorts, MAS5.0 normalized arrays, and a Bayesian approach assuming an equal amount of ABC and GCB patients. However, a classifier based on logistic regression regularized by an elastic net penalty was implemented to make the classification more adaptable to RMA normalization and one-by-one processing. This classifier proved to be quite comparable with the naive Bayes classifier over the four studied datasets confirming the strong and stable signal of the ABC/GBC subclasses of DLBCL.

Under the validation of the one-by-one method one should notice that the unclassified is treated as a class in its own right. This implies a lower accuracy compared to an approach where the unclassified are left out of the validations. The latter approach seems reasonable as changing classifications to unclassified is less serious than changing real classes. Despite the disputed properties of Cohen’s *κ* the conservative approach is retained and the issue is addressed using a Cohen’s weighted *κ* approach. Given that an idealised approach is problematic to formulate, readers are encouraged to consider the confusion matrices in the supplementary material to make an overall evaluation of the performance.

ABC/GCB, BAGS, and REGS are only a part of the GEP-based armamentarium of methods for stratifying lymphoma patients into risk groups [50–52] and it would be interesting to extend the tool to include other classification systems. For a comprehensive review see [53]. To our knowledge only a few other classification methods have been made easily accessible as either web or desktop applications. Hopefully, this research will inspire bioinformaticians and statisticians to make their cancer classification methods easily accessible for usage, speedy validation, critical reviews, and mutual inspiration.

## Conclusion

Although high throughput technologies in molecular biology have been around for almost two decades, only a few of the numerous biomarkers identified have undergone extensive validation and made it into the clinic [54]. It is our hope that making our own findings publicly available in this way will speed up validation and testing of BAGS and REGS by other researchers without having to delve into extensive bioinformatics implementations. Although hemaClass.org is still separated from the clinic we believe that a web based tool and suggestion for a clinical reference sample will bring cancer classification closer to the clinic. Hopefully, this work can also spawn interesting discussions on the clinical requirements of GEP based diagnostic and prognostic tools.

All material for reproducing this paper and its results is found at https://github.com/oncoclass/hemaclass-paper. Comments, suggestions, bug reports, and other issues are warmly welcome at https://github.com/oncoclass/hemaclass/issues or by mail to the corresponding author.

## Supporting Information

S1 Text All supplementary figures and tables, derivation of Grahams formula, detailed information on regular and one-by-one RMA normalization, and calculation of the RLE value and it’s impact on classification accuracy. (PDF)

S1 Table Confusion tables for the ABC/GCB classifiers. The columns represent cohort based normalisztion using the ABC/GCB classifier based on elastic net. The first part of the table compares Wright’s method for ABC/GCB classification with the elastic net based. In the second and third part ExLab and InLab reference based normalization is compared to cohort based normalization using the ABC/GCB classifier based on elastic net.
(PDF)

S2 Table Confusion tables for the BAGS classifier. ExLab and InLab reference based normalization are shown in the columns and cohort normalization in the rows.
(PDF)

S3 Table Confusion tables for the REGS classifiers. ExLab normalization is shown in the rows and cohort normalization in the columns.
(PDF)

S4 Table Confusion tables for the REGS classifiers. InLab normalization is shown in the rows and cohort normalization in the columns. Note, 30 samples were used as reference data and hence not present in this table.
(PDF)

S5 Table Number of probes used in the classifiers and the number of corresponding HGNC and Ensembl gene IDs. (PDF)

S6 Table Optimal thresholds for RLE IQR. (PDF)

S1 Fig Ten fold cross validation for the parameters *α* and *λ* in a logistic regression regularized by elastic net. In panels A and B the deviance is plotted against the model parameter *α* and regularization parameter *λ*, respectively. In Panel C the regularization curves are shown. Black and grey curves represent selected and non-selected probe-sets, respectively. Positive and negative coefficients indicate that high expression values for the associated gene are related to ABC and GCB, respectively. The red line indicates the model chosen through 10 fold cross validation. The gene symbols for the 20 probe-sets associated with the largest absolute coefficients in the chosen gene expression predictors are displayed in Panel C.
(TIFF)

S2 Fig Absolute value of the median (A) and IQR (B) RLE values for different RMA normalizations of the CHEPRETRO dataset and ROC curves for using these values to separate between an InLab and Exlab RMA reference (C, D). (TIFF)

S3 Fig Absolute value of the median (A) and IQR (B) RLE values for different RMA normalizations of the LLMPP R-CHOP dataset and ROC curves for using these values to separate between an InLab and Exlab RMA reference (C, D). (TIFF)

S4 Fig Absolute value of the median (A) and IQR (B) RLE values for different RMA normalizations of the LLMPP CHOP dataset and ROC curves for using these values to separate between an InLab and Exlab RMA reference (C, D). (TIFF)

S5 Fig Absolute value of the median (A) and IQR (B) RLE values for different RMA normalizations of the IDRC dataset and ROC curves for using these values to separate between an InLab and Exlab RMA reference (C, D). (TIFF)

S6 Fig Absolute value of the median (A) and IQR (B) RLE values for different RMA normalizations of the MDFCI dataset and ROC curves for using these values to separate between an InLab and Exlab RMA reference (C, D). (TIFF)

S7 Fig Proportion of samples retained (A, C, E), and accuracy (B, D, F) of BAGS classification (percent similar with cohort based) against increasing RLE IQR thresholds for different references in CHEPRETRO. The vertical line marks the suggested threshold of 0.6. (TIFF)

S8 Fig Proportion of samples retained (A, C, E), and accuracy (B, D, F) of ABC/GCB classification (percent similar with cohort based) against increasing RLE IQR thresholds for different references in CHEPRETRO. The vertical line marks the suggested threshold of 0.6. (TIFF)

S9 Fig Proportion of samples retained (A, C, E), and accuracy (B, D, F) of REGS(combined) classification (percent similar with cohort based) against increasing RLE IQR thresholds for different references in CHEPRETRO. The vertical line marks the suggested threshold of 0.6. (TIFF)

S10 Fig Proportion of samples retained (A, C, E), and accuracy (B, D, F) of BAGS classification (percent similar with cohort based) against increasing RLE IQR thresholds for different references in LLMPP R-CHOP. The vertical line marks the suggested threshold of 0.6. (TIFF)

S11 Fig Proportion of samples retained (A, C, E), and accuracy (B, D, F) of ABC/GCB classification (percent similar with cohort based) against increasing RLE IQR thresholds for different references in LLMPP R-CHOP. The vertical line marks the suggested threshold of 0.6. (TIFF)

S12 Fig Proportion of samples retained (A, C, E), and accuracy (B, D, F) of REGS(combined) classification (percent similar with cohort based) against increasing RLE IQR thresholds for different references in LLMPP R-CHOP. The vertical line marks the suggested threshold of 0.6. (TIFF)
