# Supplementary material for: hemaClass.org: Online One-By-One Microarray Normalization and Classification of Hematological Cancers for Precision Medicine
Source: PLoS One. 2016 Oct 4;11(10):e0163711. doi: 10.1371/journal.pone.0163711 (PMC5049784; doi:10.1371/journal.pone.0163711)
Supplement: S1 Text — (PDF) [file pone.0163711.s001.pdf]

SUPPLEMENTARY MATERIAL

`hemaClass.org`: Online one-by-one microarray normalization and classification of hematological cancers for precision medicine

S1 Supplementary figures and tables

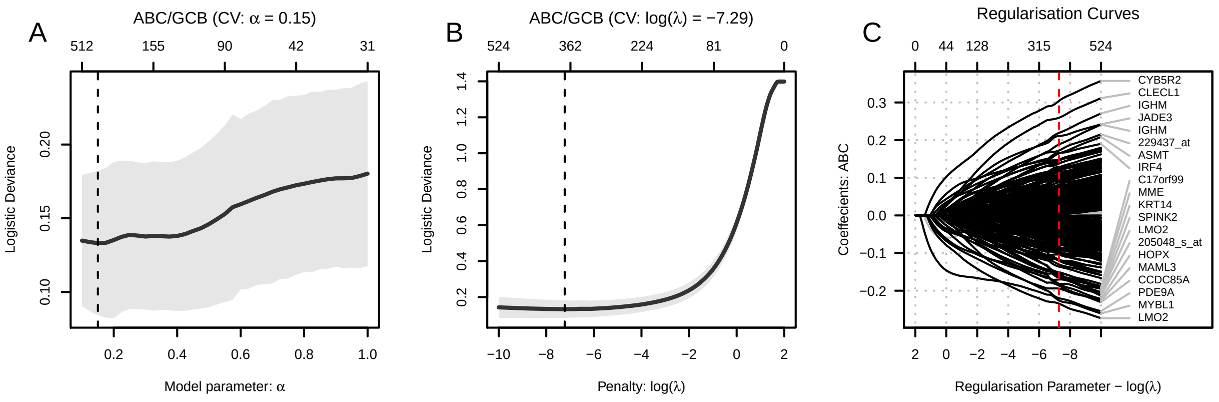

Figure S1: Ten fold cross validation for the parameters α and λ in a logistic regression regularized by elastic net. In panels A and B the deviance is plotted against the model parameter α and regularization parameter λ, respectively. In Panel C the regularization curves are shown. Black and grey curves represent selected and non-selected probe-sets, respectively. Positive and negative coefficients indicate that high expression values for the associated gene are related to ABC and GCB, respectively. The red line indicates the model chosen through 10 fold cross validation. The gene symbols for the 20 probe-sets associated with the largest absolute coefficients in the chosen gene expression predictors are displayed in Panel C.

Table S1: Confusion tables for the ABC/GCB classifiers. The columns represent cohort based normalisztion using the ABC/GCB classifier based on elastic net. The first part of the table compares Wright's method for ABC/GCB classification with the elastic net based. In the second and third part ExLab and InLab reference based normalization is compared to cohort based normalization using the ABC/GCB classifier based on elastic net.

	**CHEPRETRO**			**MDFCI**			**IDRC**			**LLMPP R-CHOP**		
	ABC	NC	GCB	ABC	NC	GCB	ABC	NC	GCB	ABC	NC	GCB
**Wright's method**												
ABC	38	2	0	28	14	0	188	24	1	90	3	0
NC	1	4	0	1	11	3	6	27	14	6	19	8
GCB	0	2	42	0	1	29	7	35	193	0	5	102
**ExLab Normalization**												
ABC	34	0	0	24	0	0	95	0	0	76	0	0
NC	5	2	0	6	4	0	102	19	0	20	6	0
GCB	0	6	42	0	22	35	4	67	208	0	21	110
**InLab Normalization**												
ABC	26	0	0	19	0	0	183	9	0	86	6	0
NC	0	5	0	0	19	0	6	63	7	0	13	4
GCB	0	1	27	0	1	22	0	9	188	0	2	92

Table S2: Confusion tables for the BAGS classifier. ExLab and InLab reference based normalization are shown in the columns and cohort normalization in the rows.

	**ExLab normalization**						**InLab normalization**					
	N	CB	CC	M	PB	UC	N	CB	CC	M	PB	UC
**CHEPRETRO**												
Naive	1	0	0	0	0	1	2	0	0	0	0	0
Centroblast	0	18	0	0	0	0	0	4	4	0	0	1
Centrocyte	0	10	11	0	5	9	0	0	25	1	0	0
Memory	0	0	0	3	0	1	0	0	0	2	0	0
Plasmablast	0	0	0	0	16	0	0	0	0	0	8	3
Unclassified	0	7	0	0	4	3	0	0	2	2	0	5
**MDFCI**												
Naive	1	1	0	1	2	3	3	0	0	1	0	2
Centroblast	0	18	0	0	1	0	0	9	0	0	0	3
Centrocyte	0	7	8	2	10	6	0	0	22	1	0	1
Memory	0	0	0	6	0	0	0	0	0	5	0	0
Plasmablast	0	0	0	0	11	0	0	0	0	0	7	0
Unclassified	0	1	0	1	7	5	1	0	0	2	1	3
**IDRC**												
Naive	0	0	3	0	6	4	12	0	0	0	1	0
Centroblast	0	16	27	0	27	22	2	62	2	0	5	12
Centrocyte	0	0	140	0	39	18	1	2	146	7	8	18
Memory	0	0	1	8	20	10	0	0	0	35	3	1
Plasmablast	0	0	1	0	74	4	0	0	0	1	76	1
Unclassified	0	0	14	0	44	17	7	0	0	9	16	38
**LLMPP R-CHOP**												
Naive	1	2	0	0	5	6	8	0	0	1	0	1
Centroblast	0	32	0	0	2	7	0	37	1	0	0	1
Centrocyte	0	5	54	0	18	12	0	1	66	1	1	4
Memory	0	0	0	5	13	4	0	0	0	22	0	0
Plasmablast	0	0	0	0	32	0	0	0	0	1	24	4
Unclassified	0	2	0	0	27	6	7	1	0	1	0	21

Table S3: Confusion tables for the REGS classifiers. ExLab normalization is shown in the rows and cohort normalization in the columns.

	CHEPRETRO			MDFCI			IDRC			LLMPP R-CHOP		
	Sen	Int	Res	Sen	Int	Res	Sen	Int	Res	Sen	Int	Res
**Cyclophosphamide**												
Sensitive	40	1	0	34	5	0	178	0	0	108	2	0
Intermediate	6	17	0	0	15	6	114	0	0	8	32	1
Resistant	0	3	22	0	1	30	203	0	0	0	15	67
**Doxorubicin**												
Sensitive	30	0	0	29	0	0	25	86	39	77	0	0
Intermediate	21	6	0	32	0	0	0	6	170	78	1	0
Resistant	0	14	18	6	12	12	0	0	169	13	43	21
**Vincristine**												
Sensitive	36	0	0	33	0	0	42	90	33	78	0	0
Intermediate	7	9	0	24	2	0	1	17	136	59	15	0
Resistant	1	10	26	1	15	16	1	3	172	11	36	34
**Combined**												
Sensitive	32	0	0	33	0	0	135	14	1	87	0	0
Intermediate	19	9	0	27	1	0	19	143	21	70	0	0
Resistant	0	13	16	3	13	14	0	27	135	16	42	18

Table S4: Confusion tables for the REGS classifiers. InLab normalization is shown in the rows and cohort normalization in the columns. Note, 30 samples were used as reference data and hence not present in this table.

	CHEPRETRO			MDFCI			IDRC			LLMPP R-CHOP		
	Sen	Int	Res	Sen	Int	Res	Sen	Int	Res	Sen	Int	Res
**Cyclophosphamide**												
Sensitive	13	10	0	26	4	0	134	32	0	89	5	0
Intermediate	0	7	10	0	10	1	3	77	29	0	27	9
Resistant	0	0	19	0	0	20	0	9	181	0	2	71
**Doxorubicin**												
Sensitive	18	2	0	19	0	0	132	7	0	50	15	0
Intermediate	0	14	2	0	21	0	24	143	3	0	55	13
Resistant	0	0	23	0	3	18	0	16	140	0	0	70
**Vincristine**												
Sensitive	18	5	0	16	6	0	127	32	0	71	0	0
Intermediate	0	10	1	0	8	9	12	83	46	9	49	0
Resistant	0	0	25	0	0	22	1	10	154	0	10	64
**Combined**												
Sensitive	19	3	0	23	1	0	125	14	0	64	12	0
Intermediate	0	11	5	0	16	0	12	148	14	0	46	10
Resistant	0	0	21	0	0	21	0	6	146	0	0	71

Classifier	nProbes	nHGNC	nEnsembl
ABC/GCB	381	291	273
BAGS	327	224	205
Vincristine Classifier	33	32	29
Vincristine Predictor	22	21	18
Cyclophosphamide Classifier	74	73	66
Cyclophosphamide Predictor	28	27	25
Doxorubicine Classifier	119	118	112
Doxorubicine Predictor	53	52	48
Combined Classifier	203	202	185
Combined Predictor	90	88	80

Table S5: Number of probes used in the classifiers and the number of corresponding HGNC and Ensembl gene IDs

S2 Graham's formula

This section derives Graham's formula which, in our context, yields the posterior probability of resistance to the combination of multiple drugs, given resistance to the individual drugs. For simplicity, the formula is derived for two drugs. The formula straightforwardly generalizes to three or more drugs.

Let C, H, and B be Bernoulli distributed random variables with probability parameter $1/2$, where $C = 1$ indicates resistance to Cyclophosphamide C, $H = 1$ indicates resistance to Doxorubicin H, and $B = 1$ indicates resistance to the combination of H and C. Conversely, C, H, and $B = 0$ indicate sensitivity towards C, H, and B, respectively. Under an assumption of conditional drug independence

$$P(C = 1, H = 1|B = 1) = P(C = 1|B = 1)P(H = 1|B = 1), \text{ and}$$
$$P(C = 1, H = 1|B = 0) = P(C = 1|B = 0)P(H = 1|B = 0)$$

we have that

$$\begin{aligned}
&P(B = 1|H = 1, C = 1)\\
&\quad = \frac{P(C = 1, H = 1, B = 1)}{P(C = 1, H = 1)}\\
&\quad = \frac{P(C = 1, H = 1|B = 1)P(B = 1)}{P(C = 1, H = 1, B = 1) + P(C = 1, H = 1, B = 0)}\\
&\quad = \frac{P(C = 1|B = 1)P(H = 1|B = 1)P(B = 1)}{P(C = 1, H = 1|B = 1)P(B = 1) + P(H = 1, C = 1|B = 0)P(B = 0)},
\end{aligned}$$

by the definition of conditional probabilities, the law of total probability, and the assumptions. From the distributional assumption on B, $P(B = 0) = P(B = 1) = 1/2$, and the above then simplifies to:

$$P(B = 1|H = 1, C = 1) = \frac{P(C = 1|B = 1)P(H = 1|B = 1)}{P(C = 1, H = 1|B = 1) + P(H = 1, C = 1|B = 0)}.$$

For notational convenience, we abbreviate $P_C = P(C = 1|B = 1)$, $P_H = P(H = 1|B = 1)$, $P_{CH} = P(B = 1|H = 1, C = 1)$. The distributional assumptions then imply:

$$\begin{aligned}
P_{CH} &= \frac{P_C P_H}{P_C P_H + P(C = 1|B = 0)P(H = 1|B = 0)}\\
&= \frac{P_C P_H}{P_C P_H + P(B = 0|C = 1)P(B = 0|H = 1)}\\
&= \frac{P_C P_H}{P_C P_H + \big(1 - P(B = 1|C = 1)\big)\big(1 - P(B = 1|H = 1)\big)}\\
&= \frac{P_C P_H}{P_C P_H + (1 - P_C)(1 - P_H)},
\end{aligned}$$

which is the two-drug equivalent to the used formula.

S3 RMA normalization

Recall that ordinary robust multichip average (RMA) pre-processing consists of three steps: (1) Background adjustment, (2) quantile normalization, and (3) summarization of probes to probe-sets, see e.g. [5, 6]. For completeness we review ordinary cohort based RMA normalization.

Background correction

In order to produce background adjusted probe intensities we will use the within array normal-exponential de-convolution scheme as implemented by the `rma.background.correct` command in the Bioconductor package `preprocessCores`, see [1, 5].

Quantile normalization

Let x_{ijk} be the $\log_2$-transformed and background adjusted cohort data, where $i = 1, \ldots, I$ index the arrays of the cohort data, $j = 1, \ldots, J$ index the probe-sets, and $k = 1, \ldots, K_j$ index the probes nested within probe-sets.

Furthermore, let G_i denote the empirical cumulative distribution function (ECDF) of the probes $\{x_{ijk}\}_{jk}$ on the i'th cohort array and F the ECDF of the across array averaged sample quantiles $\{\bar{x}_{\cdot(jk)}\}_{ij}$, where $\{x_{i(jk)}\}_{jk}$ is the order statistic of all probes on the i'th cohort array based on the lexicographic ordering of the indices $\{jk\}$. Then each data point is quantile normalized in the following way

$$\tilde{x}_{ijk} = F^{-1}(G_i(x_{ijk})),$$

where F^{-1} is calculated as the quantiles of type 2 [4]. This step is performed by the `RMA_norm` function with option `generateQuan` equal to one in the **hemaClass** package.

Summarization

For each probe-set j we let μ_{ij} represent the $\log_2$-scale expression level for array i and probeset j, α_{jk} the probe affinity effect, and the ϵ_{ijk}'s are independent identically distributed error terms with mean 0 and formulate the following linear additive model

$$\tilde{x}_{ijk} = \mu_{ij} + \alpha_{jk} + \epsilon_{ijk},$$

where $\sum_{k=0}^{n_j} \alpha_{jk} = 0$ for all probe-sets. The parameters are estimated by median polish [3]. The probe affinity estimates are denoted by $\hat{\alpha}_{jk}$.

The RMA normalized cohort data are then given by

$$\hat{x}_{ij} = \hat{\mu}_{ij}.$$

This step is performed by the `RMA_sum` function in the `hemaClass.org` package.

S4 One-by-one RMA normalization of user supplied data

Background correction

The background correction in one-by-one RMA normalization is unaltered as it is already works in a one-by-one fashion.

Quantile normalization

Let x_{ijk} be the $\log_2$-transformed and background corrected reference data, where $i = 1, \ldots, I_R$ index the arrays of the reference data, $j = 1, \ldots, J$ index the probe-sets, and $k = 1, \ldots, K_j$ index the probes. Assume x_{ijk} has been RMA normalized as described above. Similarly, let y_{ijk} be the log2-transformed and background corrected user supplied data, where $i = 1, \ldots, I_U$ index the arrays of the user supplied data, $j = 1, \ldots, J$ index the probe-sets, and $k = 1, \ldots, K_j$ index the probes. Furthermore, let H_i denote the ECDF of the user supplied data $\{y_{ijk}\}_{jk}$.

As quantile normalizer the ECDF of the background corrected reference data is used in place of the ususally applied ECDF of the mean of the sample quantiles

$$\tilde{y}_{ijk} = F^{-1}(H_i(y_{ijk})).$$

This step is performed by the `RMA_norm` function with options `generateQuan` equal to zero and `quantile` equal to the quantiles of the reference data in the **hemaClass** package.

Summarization

To mimic the RMA summarization the probe effects estimated by median polish on the reference data is subtracted all probes of the user data

$$\hat{y}_{ijk} = \tilde{y}_{ijk} - \hat{\alpha}_{jk}.$$

The pre-processed expression value for each probe-set is then estimated as the median of the associated probes.

$$\hat{y}_{ij} = \underset{k\in\{1,\dots,n_j\}}{\text{median}}\{\hat{y}_{ijk}\}.$$

S5 Classification

To ensure identical classification probabilities whether data is supplied as a cohort or one-by-one, we finally subtract the median of each probe-set in the reference from the corresponding probe-set and scale by the standard deviation of each probe-set in the reference data

$$(\hat{y}_{ij} - \hat{x}_{\cdot j})/s_{\cdot j},$$

where $\hat{x}_{\cdot j} = \text{median}_{i\in\{1,\dots,I_R\}}\{\hat{x}_{ij}\}$ and $s_{\cdot j} = \text{sd}_{i\in\{1,\dots,I_R\}}\{\hat{x}_{ij}\}$.

S6 Model control of one-by-one RMA normalization

The one-by-one RMA normalization of samples might result in biased data if the lab specific batch effects in samples are different from those in the reference. In this section we investigate the quality of data following normalization and subsequent impact on classification accuracy with the aim of validating the suitability of a chosen reference for one-by-one normalization.

Relative Log Expression

The relative log expression (RLE) is a quality measure for microarrays introduced by Bolstad et al. [2]. Using the notation from above we define the RLE for probe-set j on array i following cohort RMA normalization as:

$$\text{RLE}(\hat{y}_{ij}) = \hat{y}_{ij} - \hat{x}_{\cdot j}$$

i.e. the difference between the estimated expression for probe-set j on array i and the median expression for probe-set j in the cohort. A non-zero median RLE across probe-sets for an array thus indicates differences in the number of up- and downregulated genes, while a large interquartile range (IQR) indicates that most genes on a given array are differentially expressed [7]. Extreme values of these measures may be used to identify arrays with low-quality data. We propose that they may also be used to evaluate how well a sample resembles a given RMA reference following one-by-one normalization (how well it has been normalized), by substituting the cohort median with the RMA reference median.

RLE for separation of InLab and ExLab RMA references

For each of the five datasets used in the current study a random subset of 30 samples were extracted and set as an InLab reference. The remaining samples for each dataset were then RMA normalized as a cohort, or one-by-one against the randomly selected InLab reference or against the four other ExLab datasets. RLE values were calculated in all six scenarios and summarized as the absolute value of the median and IQR for each sample. These values are shown in panel A and B in Figs S2 to S6. We find that the RLE values in the InLab one-by-one scenarios more closely resemble the values calculated from cohort normalization than the values from ExLab one-by-one normalization. Furthermore differences between InLab and ExLab RLE values are more pronounced for the IQR of RLE than for the median. This suggests that we can aim for a method

that effectively distinguishes between InLab and ExLab reference datasets. ROC curves showing the ability of the RLE median or IQR to distinguish between an InLab or ExLab reference were calculated and plotted using the pROC package version 1.8 [8] in R, as shown in panels C and D in Figs S2 to S6. The area under the curve (AUC) for the ROC curves confirm the superiority of the RLE IQR for distinguishing between a "correct" reference and a "wrong" reference, i.e. in most cases we observe a higher AUC when using the IQR instead of the median RLE. However, for CHEPRETRO the RLE IQR values for the LLMPP R-CHOP and MDFCI reference are not as clearly separated from the InLab values as IDRC and LLMPP CHOP (Fig S2D).

Using Youden's index Youden [9], which maximizes the sum of sensitivity and specificity, we calculated the optimal threshold for each ROC curve based on the RLE IQR as shown in Table S6. Calculating the median RLE IQR across all datasets and one-by-one RMA references gives a value of 0.62. By rounding we set a conservative threshold of 0.6 for reference normalized samples, i.e. if the RLE IQR exceeds this value the array is expected to have been normalized incorrectly.

RLE IQR vs Classification accuracy

The results in Supplementary Section showed that the RLE IQR can be used to determine if samples have been normalized against an Inlab or ExLab reference. In this section we compare the RLE IQR to the classification accuracy, to see if samples with higher RLE IQR values result in a higher proportion of misclassifications when compared to cohort normalized data.

The CHEPRETRO dataset was one-by-one normalized against an InLab reference, the LLMPP CHOP reference, and the LLMPP R-CHOP reference, and the LLMPP R-CHOP dataset was one-by-one normalized against an InLab reference, the LLMPP CHOP reference, and the CHEPRETRO reference. RLE IQR values were calculated and the proportion of samples below a given threshold and the accuracy (proportion of samples with similar classification in cohort normalized data) were calculated for increasing values of the RLE IQR. This was done for ABC/GCB, BAGS and REGS classification. Results are shown in Figs S7 to S12.

For CHEPRETRO we saw that most samples were retained at the suggested RLE IQR value of 0.6 when samples were normalized against the InLab reference, and a tendency towards higher classification accuracy for samples with low RLE IQR. When normalizing CHEPRETRO against LLMPP R-CHOP the RLE IQR value of 0.6 only excludes a small proportion of the samples and no clear differences in the accuracy is observed when including samples with higher RLE IQR. For LLMPP CHOP normalization most samples are removed at the suggested value, and a high accuracy for the few remaining samples are seen for BAGS and ABC/GCB classification while an accuracy of zero is seen for REGS. Comparing the overall classification accuracy for CHEPRETRO samples normalized against either the LLMPP CHOP or LLMPP R-CHOP reference the accuracy is higher, and closer to the InLab level, in the LLMPP R-CHOP scenario where RLE IQR values on average are lower

For the LLMPP R-CHOP dataset most InLab reference normalized samples were retained at the suggested value of 0.6, but lower RLE IQR values did not give higher classification accuracies. Most samples normalized against the CHEPRETRO or CHOP reference are excluded at an RLE IQR of 0.6, but there is no clear indication of higher accuracies for samples with RLE IQR below the threshold.

Looking at the results as a whole, a low IQR in itself does not guarantee a higher classification accuracy, but we notice that excluding samples with an RLE IQR value above 0.6 ensures that we either exclude most samples normalized against an ExLab reference or retain a classification accuracy at almost the same level as for the InLab reference. We therefore recommend removing samples from the analysis if the RLE IQR exceeds a value of 0.6.

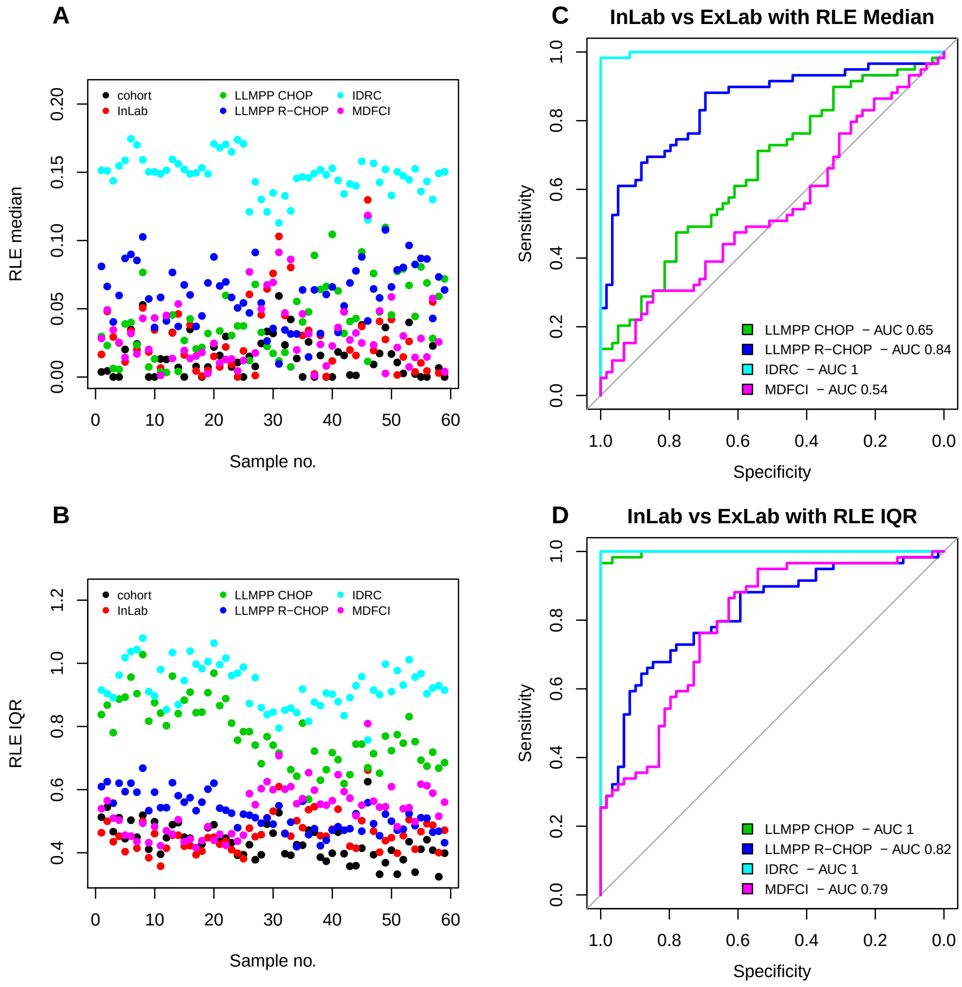

Figure S2: Absolute value of the median (A) and IQR (B) RLE values for different RMA normalizations of the CHEPRETRO dataset and ROC curves for using these values to separate between an InLab and Exlab RMA reference (C,D)

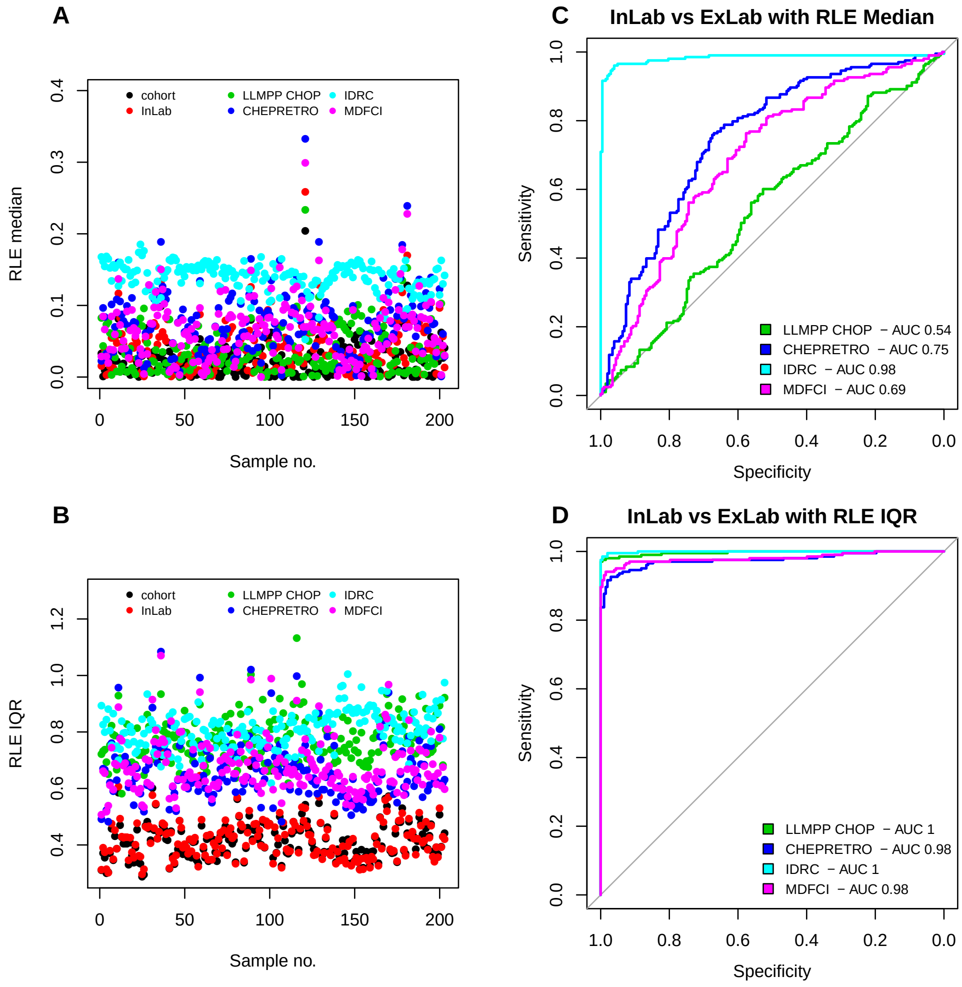

Figure S3: Absolute value of the median (A) and IQR (B) RLE values for different RMA normalizations of the LLMPP R-CHOP dataset and ROC curves for using these values to separate between an InLab and Exlab RMA reference (C,D)

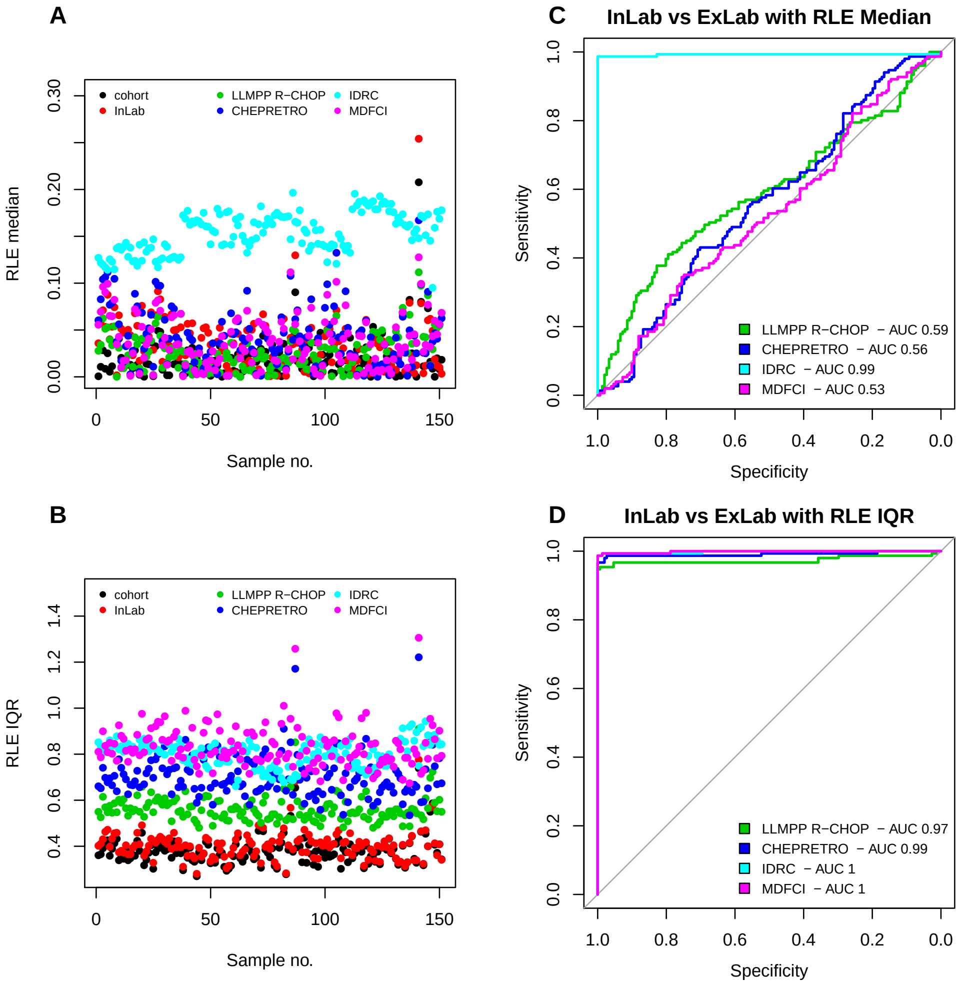

Figure S4: Absolute value of the median (A) and IQR (B) RLE values for different RMA normalizations of the LLMPP CHOP dataset and ROC curves for using these values to separate between an InLab and Exlab RMA reference (C,D)

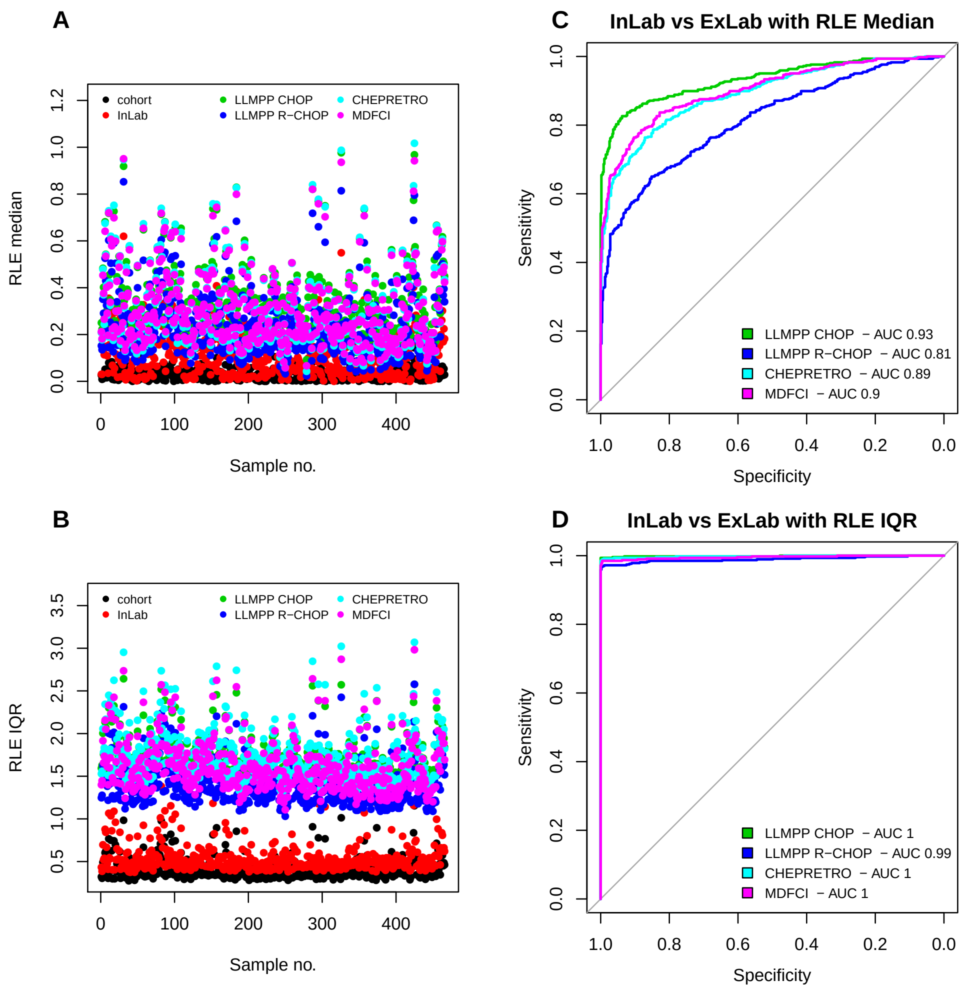

Figure S5: Absolute value of the median (A) and IQR (B) RLE values for different RMA normalizations of the IDRC dataset and ROC curves for using these values to separate between an InLab and Exlab RMA reference (C,D)

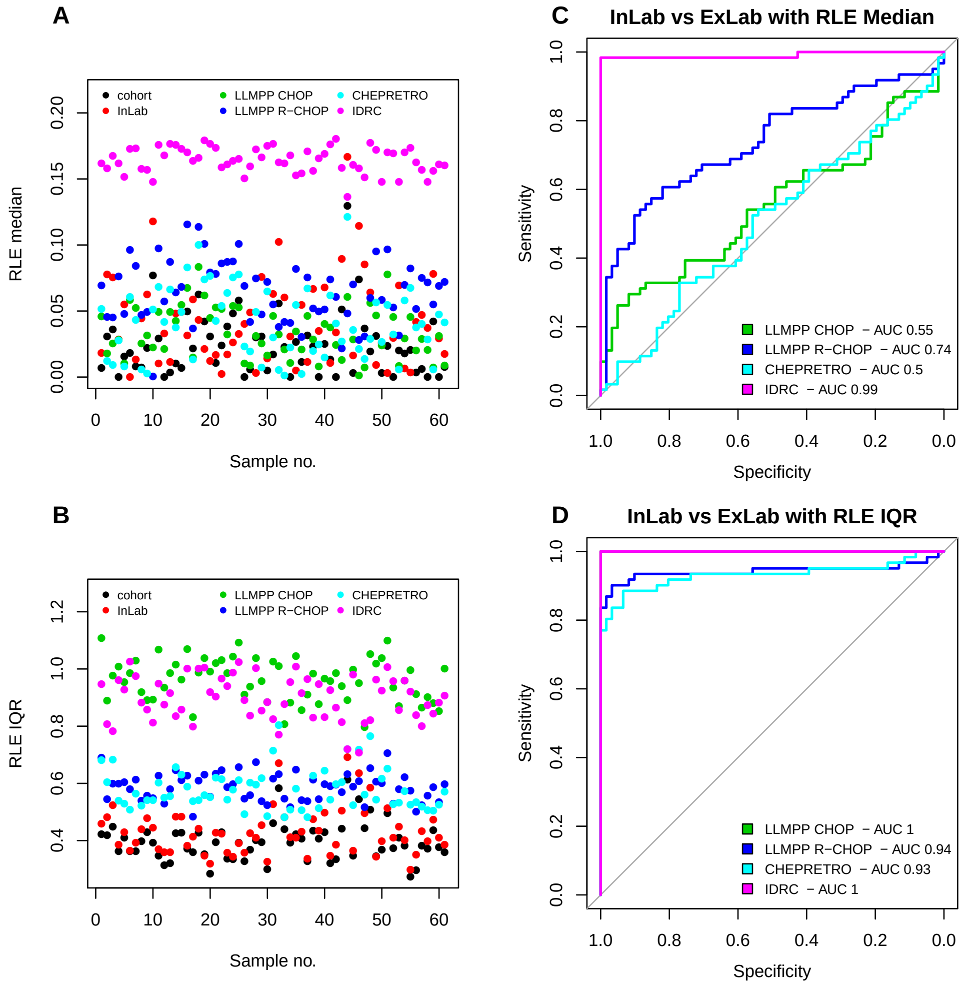

Figure S6: Absolute value of the median (A) and IQR (B) RLE values for different RMA normalizations of the MDFCI dataset and ROC curves for using these values to separate between an InLab and Exlab RMA reference (C,D)

Dataset	RMA reference	Threshold	Sensitivity	Specificity
CHEPRETRO	LLMPP CHOP	0.60	0.95	0.98
CHEPRETRO	LLMPP R-CHOP	0.48	0.58	0.85
CHEPRETRO	IDRC	0.72	1.00	1.00
CHEPRETRO	MDFCI	0.42	0.27	1.00
LLMPP R-CHOP	LLMPPP CHOP	0.61	0.98	1.00
LLMPP R-CHOP	CHEPRETRO	0.52	0.93	0.97
LLMPP R-CHOP	IDRC	0.68	0.99	1.00
LLMPP R-CHOP	MDFCI	0.53	0.94	0.99
LLMPP CHOP	LLMPPP R-CHOP	0.48	0.95	0.99
LLMPP CHOP	CHEPRETRO	0.51	0.97	1.00
LLMPP CHOP	IDRC	0.62	0.99	1.00
LLMPP CHOP	MDFCI	0.62	0.99	1.00
IDRC	CHOP	1.39	0.99	1.00
IDRC	RCHOP	1.08	0.97	1.00
IDRC	CHEP	1.32	0.99	1.00
IDRC	MDFCI	1.19	0.98	1.00
MDFCI	LLMPP CHOP	0.74	1.00	1.00
MDFCI	LLMPP R-CHOP	0.51	0.90	0.97
MDFCI	CHEPRETRO	0.50	0.89	0.93
MDFCI	IDRC	0.70	1.00	1.00
Median	-	0.62	0.97	1.00

Table S6: Optimal thresholds for RLE IQR

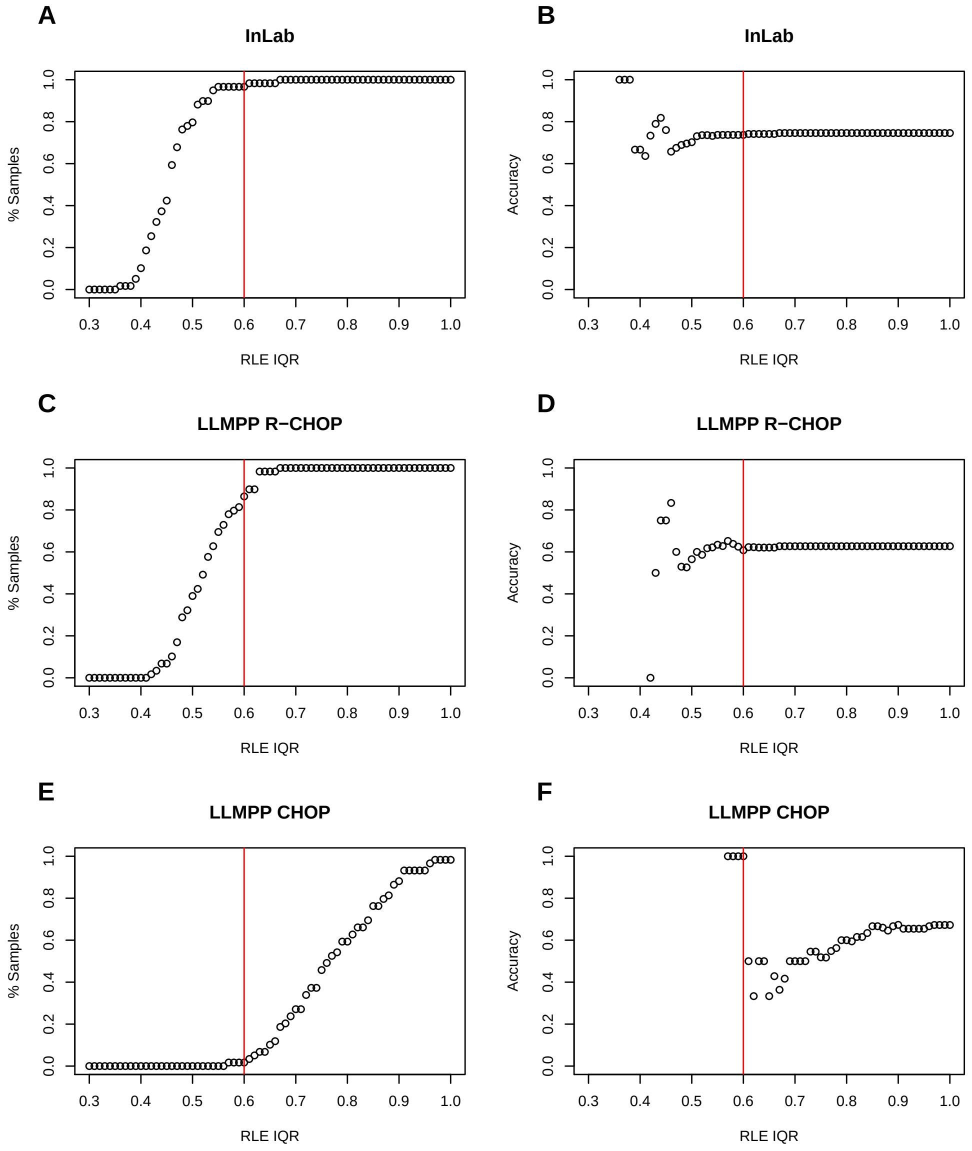

Figure S7: Proportion samples retained (A,C,E) and accuracy (B,D,F) of BAGS classification (percent similar with cohort based) against increasing RLE IQR thresholds for different references in CHEPRETRO. The vertical line marks the suggested threshold of 0.6

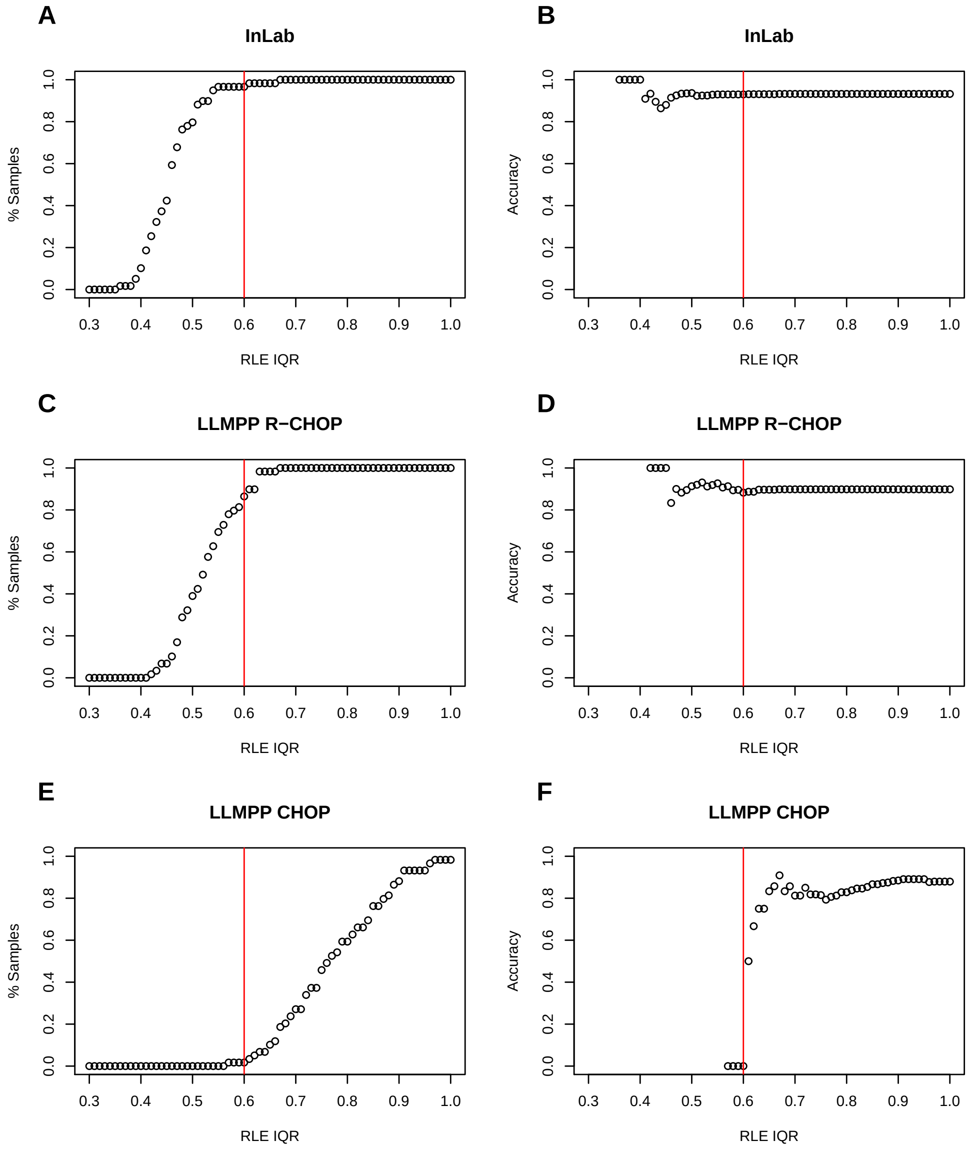

Figure S8: Proportion samples retained (A,C,E)and accuracy (B,D,F) of ABC/GCB classification (percent similar with cohort based) against increasing RLE IQR thresholds for different references in CHEPRETRO. The vertical line marks the suggested threshold of 0.6

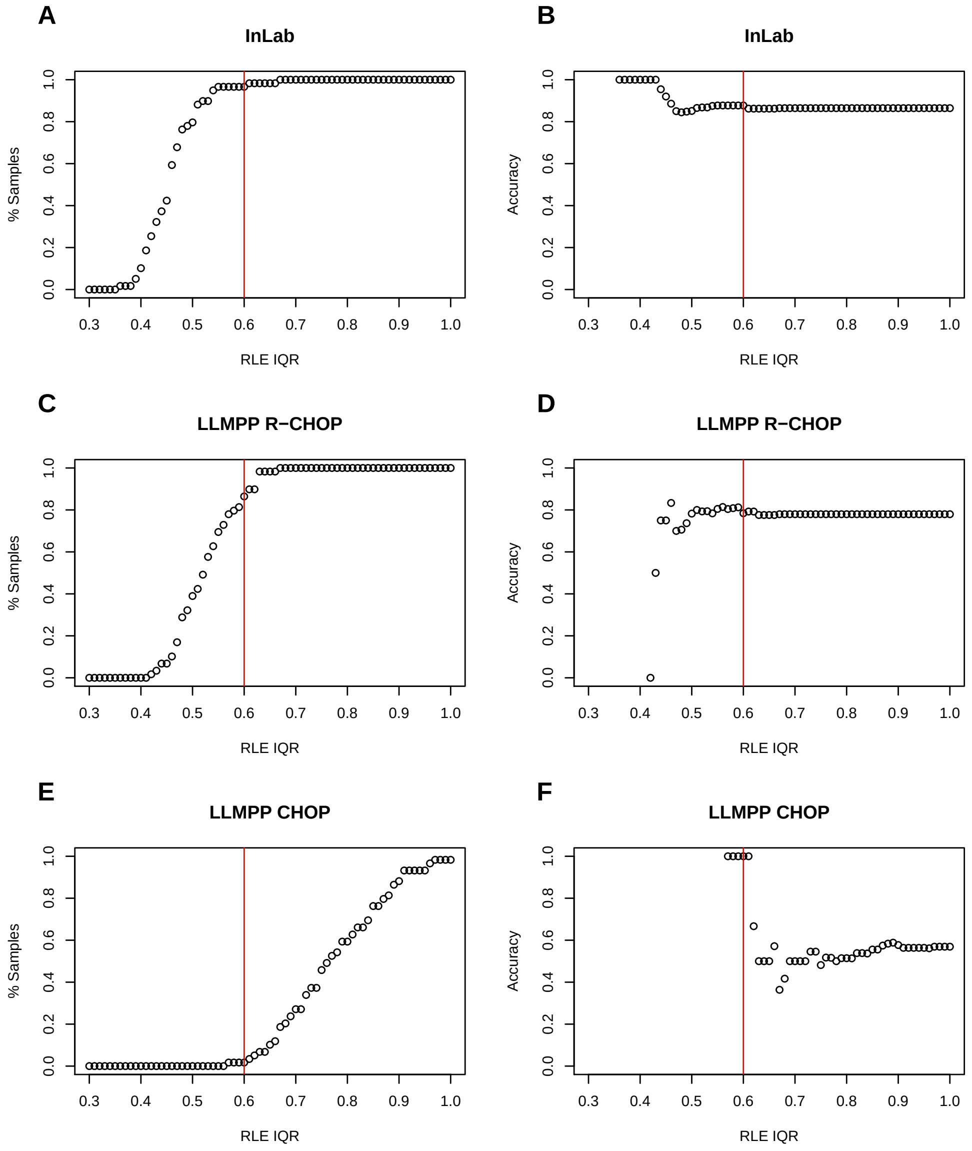

Figure S9: Proportion samples retained (A,C,E) and accuracy (B,D,F) of REGS(combined) classification (percent similar with cohort based) against increasing RLE IQR thresholds for different references in CHEP-RETRO. The vertical line marks the suggested threshold of 0.6

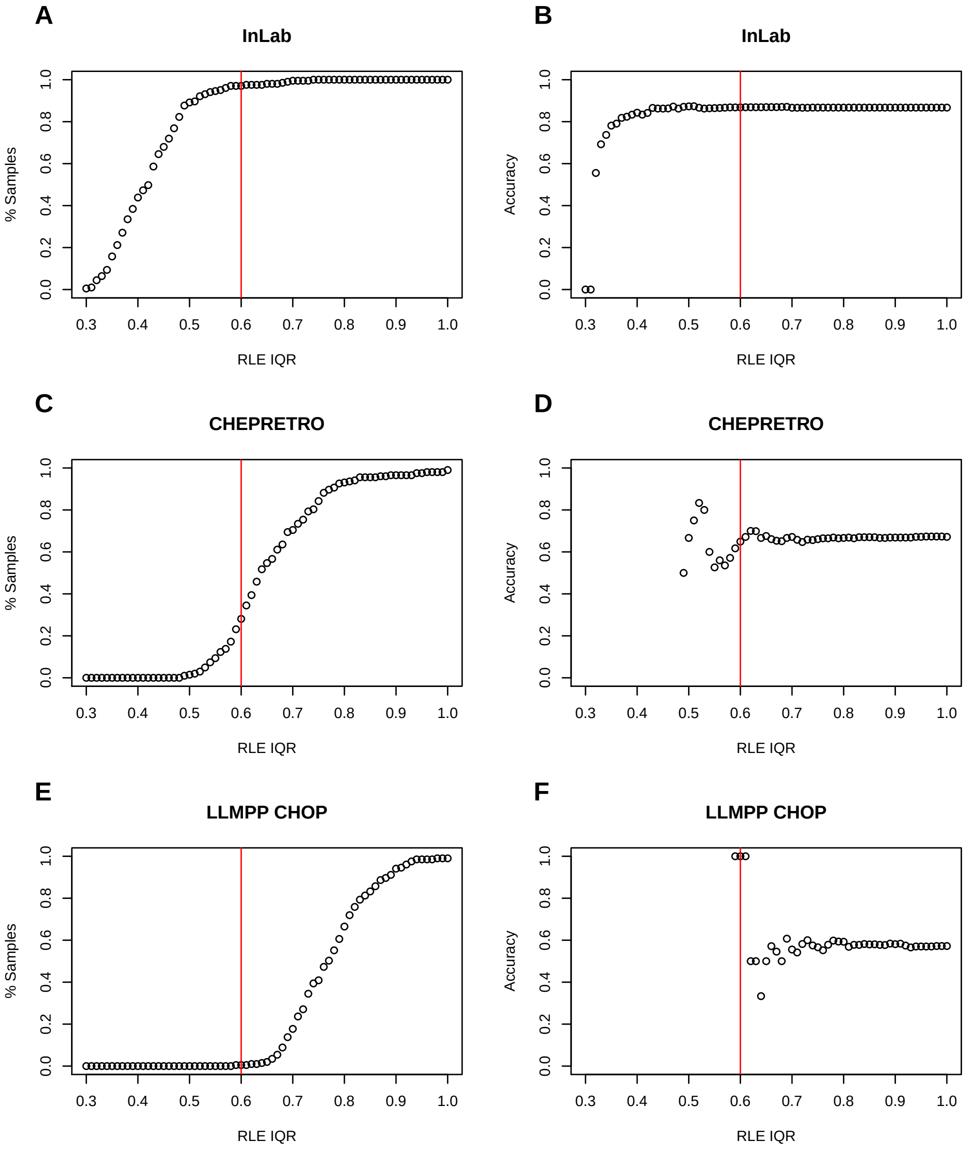

Figure S10: Proportion samples retained (A,C,E) and accuracy (B,D,F) of BAGS classification (percent similar with cohort based) against increasing RLE IQR thresholds for different references in LLMPP R-CHOP. The vertical line marks the suggested threshold of 0.6

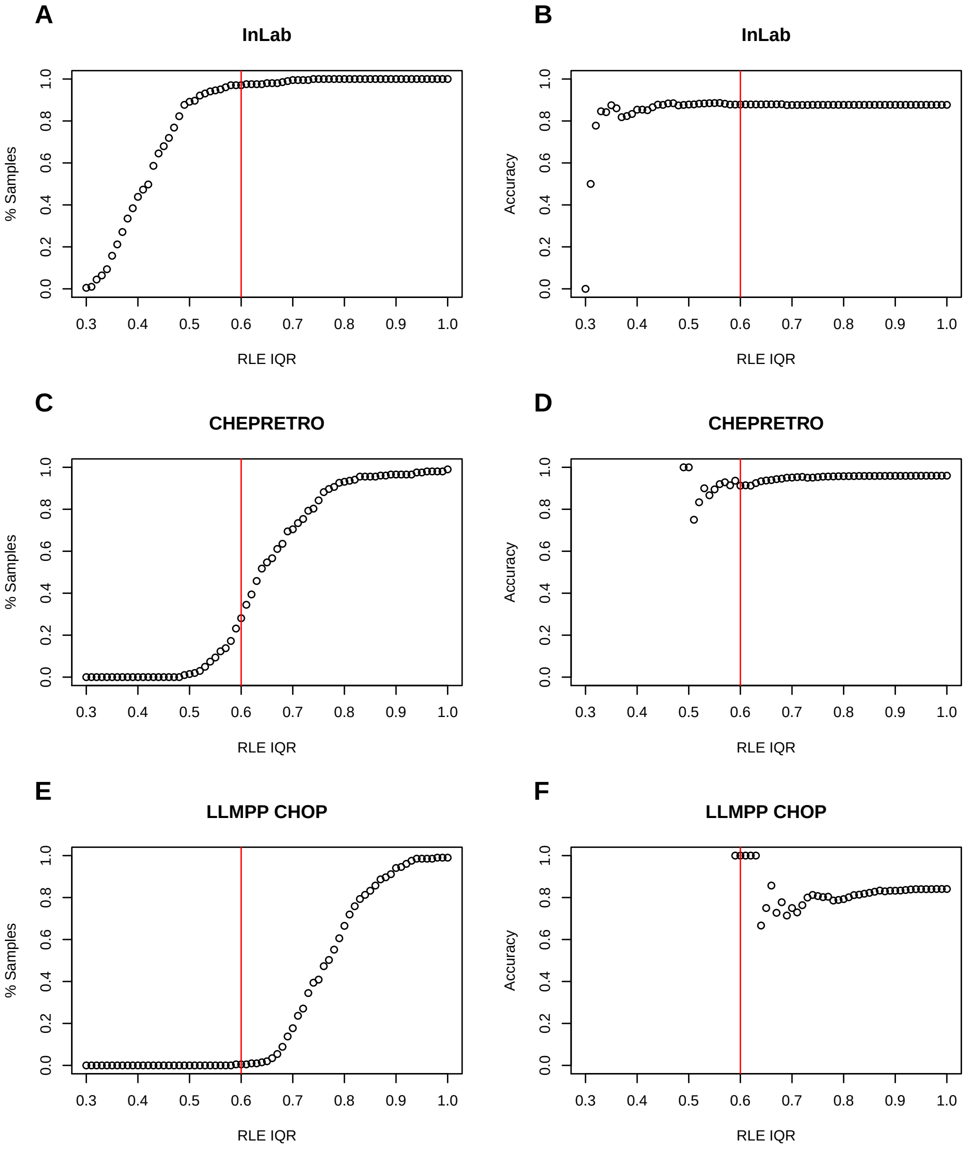

Figure S11: Proportion samples retained (A,C,E) and accuracy (B,D,F) of ABC/GCB classification (percent similar with cohort based) against increasing RLE IQR thresholds for different references in LLMPP R-CHOP. The vertical line marks the suggested threshold of 0.6

Figure S12: Proportion samples retained (A,C,E) and accuracy (B,D,F) of REGS(combined) classification (percent similar with cohort based) against increasing RLE IQR thresholds for different references in LLMPP R-CHOP. The vertical line marks the suggested threshold of 0.6

References

[1] Benjamin Milo Bolstad. *Low-level Analysis of High-Density Oligonucleotide Array Data: Background, Normalization and Summarization.* PhD thesis, University of California, Berkeley, 2004.

[2] B.M. Bolstad, F. Collin, K.M. Simpson, R.A. Irizarry, and T.P. Speed. Experimental Design and Low-Level Analysis of Microarray Data. *International Review of Neurobiology*, 60:25–58, 2004. doi: 10.1016/S0074-7742(04)60002-X.

[3] Daniel Holder, Richard F Raubertas, V Bill Pikounis, Vladimir Svetnik, and Keith Soper. Statistical analysis of high density oligonucleotide arrays: A safer approach. In *GeneLogic Workshop on Low Level Analysis of Affymetrix GeneChip Data*, 2001.

[4] Rob J Hyndman and Yanan Fan. Sample quantiles in statistical packages. *The American Statistician*, 50(4):361–365, 1996.

[5] Rafael A. Irizarry, Benjamin M. Bolstad, Francois Collin, Leslie M. Cope, Bridget Hobbs, and Terence P. Speed. Summaries of Affymetrix GeneChip probe level data. *Nucleic Acids Research*, 31(4):e15, 2003. doi: 10.1093/nar/gng015.

[6] Rafael A. Irizarry, Bridget Hobbs, Francois Collin, Yasmin D Beazer-Barclay, Kristen J Antonellis, U Scherf, and Terence P Speed. Exploration, normalization, and summaries of high density oligonucleotide array probe level data. *Biostatistics*, 4(2):249–264, 2003. doi: 10.1093/biostatistics/4.2.249.

[7] Matthew N McCall, Peter N Murakami, Margus Lukk, Wolfgang Huber, and Rafael a Irizarry. Assessing affymetrix GeneChip microarray quality. *BMC bioinformatics*, 12(1):137, 2011. doi: 10.1186/1471-2105-12-137.

[8] Xavier Robin, Natacha Turck, Alexandre Hainard, Natalia Tiberti, Frdrique Lisacek, Jean-Charles Sanchez, and Markus Mller. proc: an open-source package for r and s+ to analyze and compare roc curves. *BMC Bioinformatics*, 12:77, 2011. doi: 10.1186/1471-2105-12-77.

[9] W. J. Youden. Index for rating diagnostic tests. *Cancer*, 3(1):32–35, 1950. doi: 10.1002/1097-0142(1950)3:1⟨32::AID-CNCR2820030106⟩3.0.CO;2-3.
