# Supplementary material for: hemaClass.org: Online One-By-One Microarray Normalization and Classification of Hematological Cancers for Precision Medicine
Source: PLoS One. 2016 Oct 4;11(10):e0163711. doi: 10.1371/journal.pone.0163711 (PMC5049784; doi:10.1371/journal.pone.0163711)
Supplement: S1 Table — The columns represent cohort based normalisztion using the ABC/GCB classifier based on elastic net. The first part of the table compares Wright’s method for ABC/GCB classification with the elastic net based. In the second and third part ExLab and InLab reference based normalization is compared to cohort based normalization using the ABC/GCB classifier based on elastic net. (PDF) [file pone.0163711.s002.pdf]

Table S1: Confusion tables for the ABC/GCB classifiers. The columns represent cohort based normalisztion using the ABC/GCB classifier based on elastic net. The first part of the table compares Wright's method for ABC/GCB classification with the elastic net based. In the second and third part ExLab and InLab reference based normalization is compared to cohort based normalization using the ABC/GCB classifier based on elastic net.

	**CHEPRETRO**			**MDFCI**			**IDRC**			**LLMPP R-CHOP**		
	ABC	NC	GCB	ABC	NC	GCB	ABC	NC	GCB	ABC	NC	GCB
**Wright's method**												
ABC	38	2	0	28	14	0	188	24	1	90	3	0
NC	1	4	0	1	11	3	6	27	14	6	19	8
GCB	0	2	42	0	1	29	7	35	193	0	5	102
**ExLab Normalization**												
ABC	34	0	0	24	0	0	95	0	0	76	0	0
NC	5	2	0	6	4	0	102	19	0	20	6	0
GCB	0	6	42	0	22	35	4	67	208	0	21	110
**InLab Normalization**												
ABC	26	0	0	19	0	0	183	9	0	86	6	0
NC	0	5	0	0	19	0	6	63	7	0	13	4
GCB	0	1	27	0	1	22	0	9	188	0	2	92
