# Supplementary material for: hemaClass.org: Online One-By-One Microarray Normalization and Classification of Hematological Cancers for Precision Medicine
Source: PLoS One. 2016 Oct 4;11(10):e0163711. doi: 10.1371/journal.pone.0163711 (PMC5049784; doi:10.1371/journal.pone.0163711)
Supplement: S2 Table — ExLab and InLab reference based normalization are shown in the columns and cohort normalization in the rows. (PDF) [file pone.0163711.s003.pdf]

Table S2: Confusion tables for the BAGS classifier. ExLab and InLab reference based normalization are shown in the columns and cohort normalization in the rows.

	**ExLab normalization**						**InLab normalization**					
	N	CB	CC	M	PB	UC	N	CB	CC	M	PB	UC
**CHEPRETRO**												
Naive	1	0	0	0	0	1	2	0	0	0	0	0
Centroblast	0	18	0	0	0	0	0	4	4	0	0	1
Centrocyte	0	10	11	0	5	9	0	0	25	1	0	0
Memory	0	0	0	3	0	1	0	0	0	2	0	0
Plasmablast	0	0	0	0	16	0	0	0	0	0	8	3
Unclassified	0	7	0	0	4	3	0	0	2	2	0	5
**MDFCI**												
Naive	1	1	0	1	2	3	3	0	0	1	0	2
Centroblast	0	18	0	0	1	0	0	9	0	0	0	3
Centrocyte	0	7	8	2	10	6	0	0	22	1	0	1
Memory	0	0	0	6	0	0	0	0	0	5	0	0
Plasmablast	0	0	0	0	11	0	0	0	0	0	7	0
Unclassified	0	1	0	1	7	5	1	0	0	2	1	3
**IDRC**												
Naive	0	0	3	0	6	4	12	0	0	0	1	0
Centroblast	0	16	27	0	27	22	2	62	2	0	5	12
Centrocyte	0	0	140	0	39	18	1	2	146	7	8	18
Memory	0	0	1	8	20	10	0	0	0	35	3	1
Plasmablast	0	0	1	0	74	4	0	0	0	1	76	1
Unclassified	0	0	14	0	44	17	7	0	0	9	16	38
**LLMPP R-CHOP**												
Naive	1	2	0	0	5	6	8	0	0	1	0	1
Centroblast	0	32	0	0	2	7	0	37	1	0	0	1
Centrocyte	0	5	54	0	18	12	0	1	66	1	1	4
Memory	0	0	0	5	13	4	0	0	0	22	0	0
Plasmablast	0	0	0	0	32	0	0	0	0	1	24	4
Unclassified	0	2	0	0	27	6	7	1	0	1	0	21
