# Supplementary material for: hemaClass.org: Online One-By-One Microarray Normalization and Classification of Hematological Cancers for Precision Medicine
Source: PLoS One. 2016 Oct 4;11(10):e0163711. doi: 10.1371/journal.pone.0163711 (PMC5049784; doi:10.1371/journal.pone.0163711)
Supplement: S3 Table — ExLab normalization is shown in the rows and cohort normalization in the columns. (PDF) [file pone.0163711.s004.pdf]

Table S3: Confusion tables for the REGS classifiers. ExLab normalization is shown in the rows and cohort normalization in the columns.

	CHEPRETRO			MDFCI			IDRC			LLMPP R-CHOP		
	Sen	Int	Res	Sen	Int	Res	Sen	Int	Res	Sen	Int	Res
**Cyclophosphamide**												
Sensitive	40	1	0	34	5	0	178	0	0	108	2	0
Intermediate	6	17	0	0	15	6	114	0	0	8	32	1
Resistant	0	3	22	0	1	30	203	0	0	0	15	67
**Doxorubicin**												
Sensitive	30	0	0	29	0	0	25	86	39	77	0	0
Intermediate	21	6	0	32	0	0	0	6	170	78	1	0
Resistant	0	14	18	6	12	12	0	0	169	13	43	21
**Vincristine**												
Sensitive	36	0	0	33	0	0	42	90	33	78	0	0
Intermediate	7	9	0	24	2	0	1	17	136	59	15	0
Resistant	1	10	26	1	15	16	1	3	172	11	36	34
**Combined**												
Sensitive	32	0	0	33	0	0	135	14	1	87	0	0
Intermediate	19	9	0	27	1	0	19	143	21	70	0	0
Resistant	0	13	16	3	13	14	0	27	135	16	42	18
