# Supplementary material for: hemaClass.org: Online One-By-One Microarray Normalization and Classification of Hematological Cancers for Precision Medicine
Source: PLoS One. 2016 Oct 4;11(10):e0163711. doi: 10.1371/journal.pone.0163711 (PMC5049784; doi:10.1371/journal.pone.0163711)
Supplement: S4 Table — InLab normalization is shown in the rows and cohort normalization in the columns. Note, 30 samples were used as reference data and hence not present in this table. (PDF) [file pone.0163711.s005.pdf]

Table S4: Confusion tables for the REGS classifiers. InLab normalization is shown in the rows and cohort normalization in the columns. Note, 30 samples were used as reference data and hence not present in this table.

	**CHEPRETRO**			**MDFCI**			**IDRC**			**LLMPP R-CHOP**		
	Sen	Int	Res	Sen	Int	Res	Sen	Int	Res	Sen	Int	Res
**Cyclophosphamide**												
Sensitive	13	10	0	26	4	0	134	32	0	89	5	0
Intermediate	0	7	10	0	10	1	3	77	29	0	27	9
Resistant	0	0	19	0	0	20	0	9	181	0	2	71
**Doxorubicin**												
Sensitive	18	2	0	19	0	0	132	7	0	50	15	0
Intermediate	0	14	2	0	21	0	24	143	3	0	55	13
Resistant	0	0	23	0	3	18	0	16	140	0	0	70
**Vincristine**												
Sensitive	18	5	0	16	6	0	127	32	0	71	0	0
Intermediate	0	10	1	0	8	9	12	83	46	9	49	0
Resistant	0	0	25	0	0	22	1	10	154	0	10	64
**Combined**												
Sensitive	19	3	0	23	1	0	125	14	0	64	12	0
Intermediate	0	11	5	0	16	0	12	148	14	0	46	10
Resistant	0	0	21	0	0	21	0	6	146	0	0	71
