# Supplementary material for: hemaClass.org: Online One-By-One Microarray Normalization and Classification of Hematological Cancers for Precision Medicine
Source: PLoS One. 2016 Oct 4;11(10):e0163711. doi: 10.1371/journal.pone.0163711 (PMC5049784; doi:10.1371/journal.pone.0163711)
Supplement: S5 Table — (PDF) [file pone.0163711.s006.pdf]

| Classifier | nProbes | nHGNC | nEnsembl |
|---|---|---|---|
| ABC/GCB | 381 | 291 | 273 |
| BAGS | 327 | 224 | 205 |
| Vincristine Classifier | 33 | 32 | 29 |
| Vincristine Predictor | 22 | 21 | 18 |
| Cyclophosphamide Classifier | 74 | 73 | 66 |
| Cyclophosphamide Predictor | 28 | 27 | 25 |
| Doxorubicine Classifier | 119 | 118 | 112 |
| Doxorubicine Predictor | 53 | 52 | 48 |
| Combined Classifier | 203 | 202 | 185 |
| Combined Predictor | 90 | 88 | 80 |

Table S5: Number of probes used in the classifiers and the number of corresponding HGNC and Ensembl gene IDs
