# Supplementary material for: hemaClass.org: Online One-By-One Microarray Normalization and Classification of Hematological Cancers for Precision Medicine
Source: PLoS One. 2016 Oct 4;11(10):e0163711. doi: 10.1371/journal.pone.0163711 (PMC5049784; doi:10.1371/journal.pone.0163711)
Supplement: S6 Table — (PDF) [file pone.0163711.s007.pdf]

Dataset	RMA reference	Threshold	Sensitivity	Specificity
CHEPRETRO	LLMPP CHOP	0.60	0.95	0.98
CHEPRETRO	LLMPP R-CHOP	0.48	0.58	0.85
CHEPRETRO	IDRC	0.72	1.00	1.00
CHEPRETRO	MDFCI	0.42	0.27	1.00
LLMPP R-CHOP	LLMPPP CHOP	0.61	0.98	1.00
LLMPP R-CHOP	CHEPRETRO	0.52	0.93	0.97
LLMPP R-CHOP	IDRC	0.68	0.99	1.00
LLMPP R-CHOP	MDFCI	0.53	0.94	0.99
LLMPP CHOP	LLMPPP R-CHOP	0.48	0.95	0.99
LLMPP CHOP	CHEPRETRO	0.51	0.97	1.00
LLMPP CHOP	IDRC	0.62	0.99	1.00
LLMPP CHOP	MDFCI	0.62	0.99	1.00
IDRC	CHOP	1.39	0.99	1.00
IDRC	RCHOP	1.08	0.97	1.00
IDRC	CHEP	1.32	0.99	1.00
IDRC	MDFCI	1.19	0.98	1.00
MDFCI	LLMPP CHOP	0.74	1.00	1.00
MDFCI	LLMPP R-CHOP	0.51	0.90	0.97
MDFCI	CHEPRETRO	0.50	0.89	0.93
MDFCI	IDRC	0.70	1.00	1.00
Median	-	0.62	0.97	1.00

Table S6: Optimal thresholds for RLE IQR
