# Supplementary figures and images for: hemaClass.org: Online One-By-One Microarray Normalization and Classification of Hematological Cancers for Precision Medicine
Source: PLoS One. 2016 Oct 4;11(10):e0163711. doi: 10.1371/journal.pone.0163711 (PMC5049784; doi:10.1371/journal.pone.0163711)

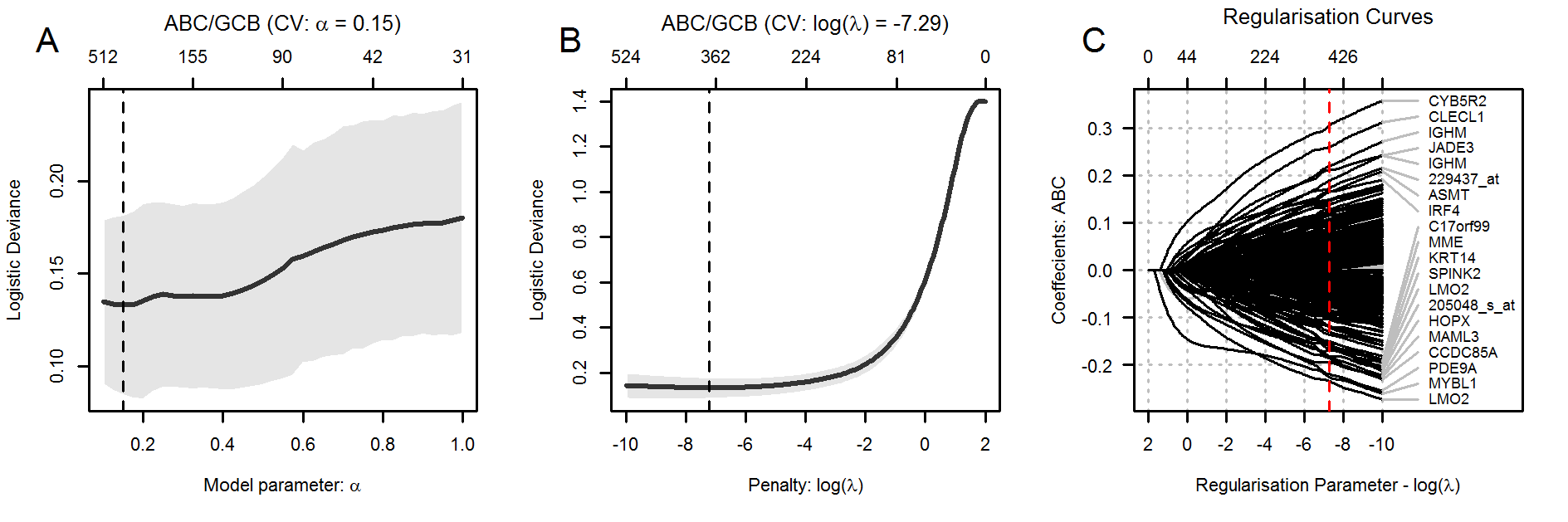

Supplement: S1 Fig — In panels A and B the deviance is plotted against the model parameter α and regularization parameter λ, respectively. In Panel C the regularization curves are shown. Black and grey curves represent selected and non-selected probe-sets, respectively. Positive and negative coefficients indicate that high expression values for the associated gene are related to ABC and GCB, respectively. The red line indicates the model chosen through 10 fold cross validation. The gene symbols for the 20 probe-sets associated with the largest absolute coefficients in the chosen gene expression predictors are displayed in Panel C. (TIFF) [file pone.0163711.s008.tiff]

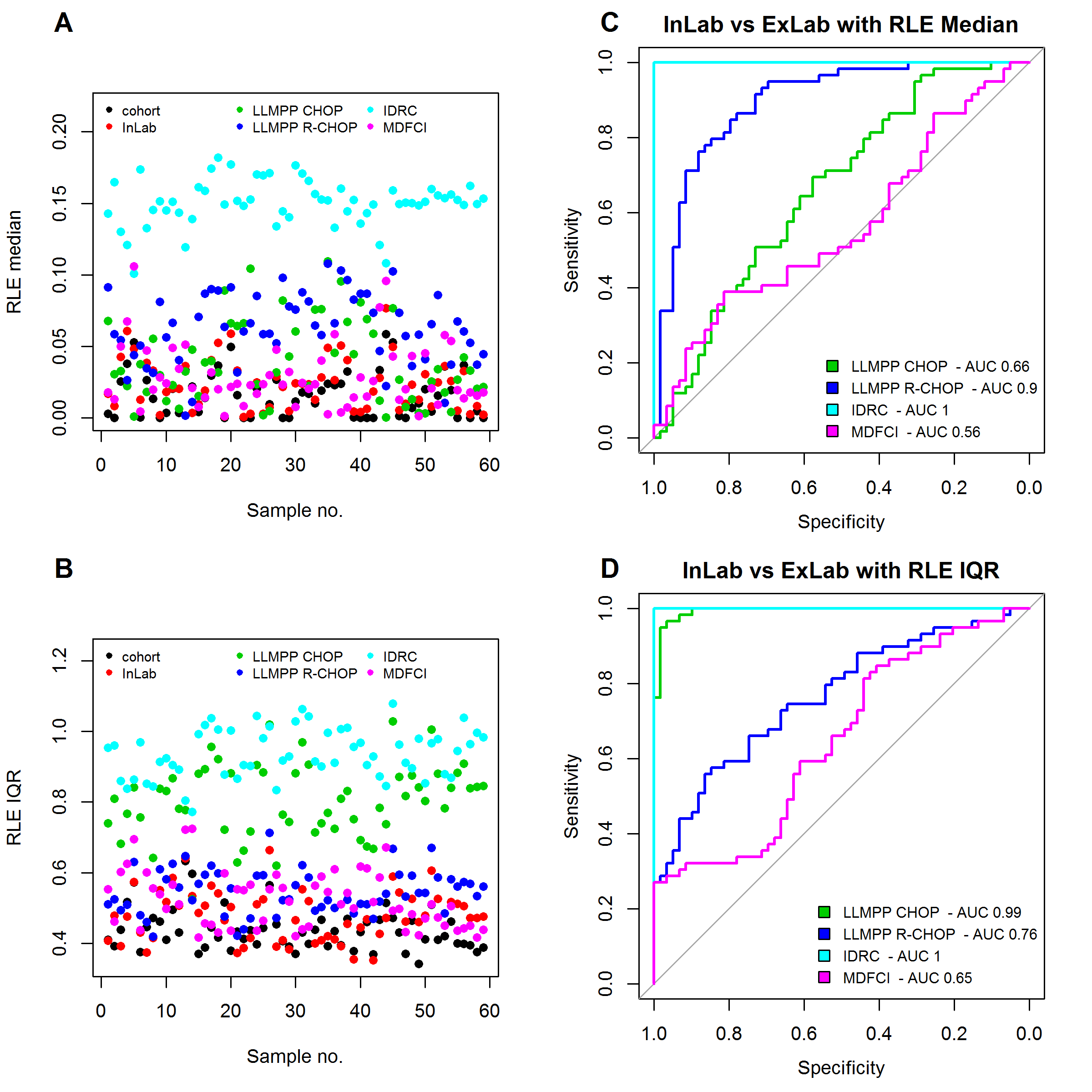

Supplement: S2 Fig — (TIFF) [file pone.0163711.s009.tiff]

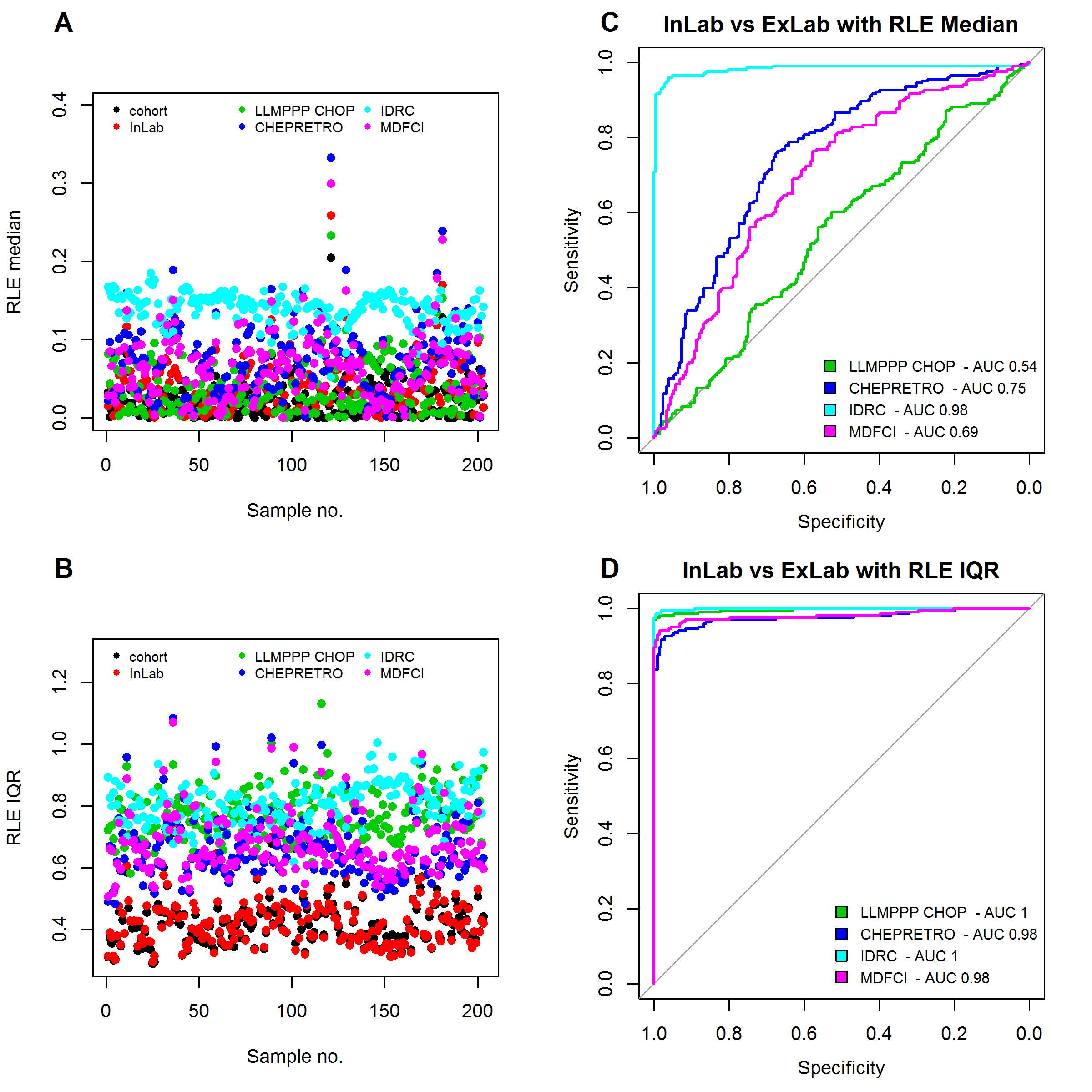

Supplement: S3 Fig — (TIFF) [file pone.0163711.s010.tiff]

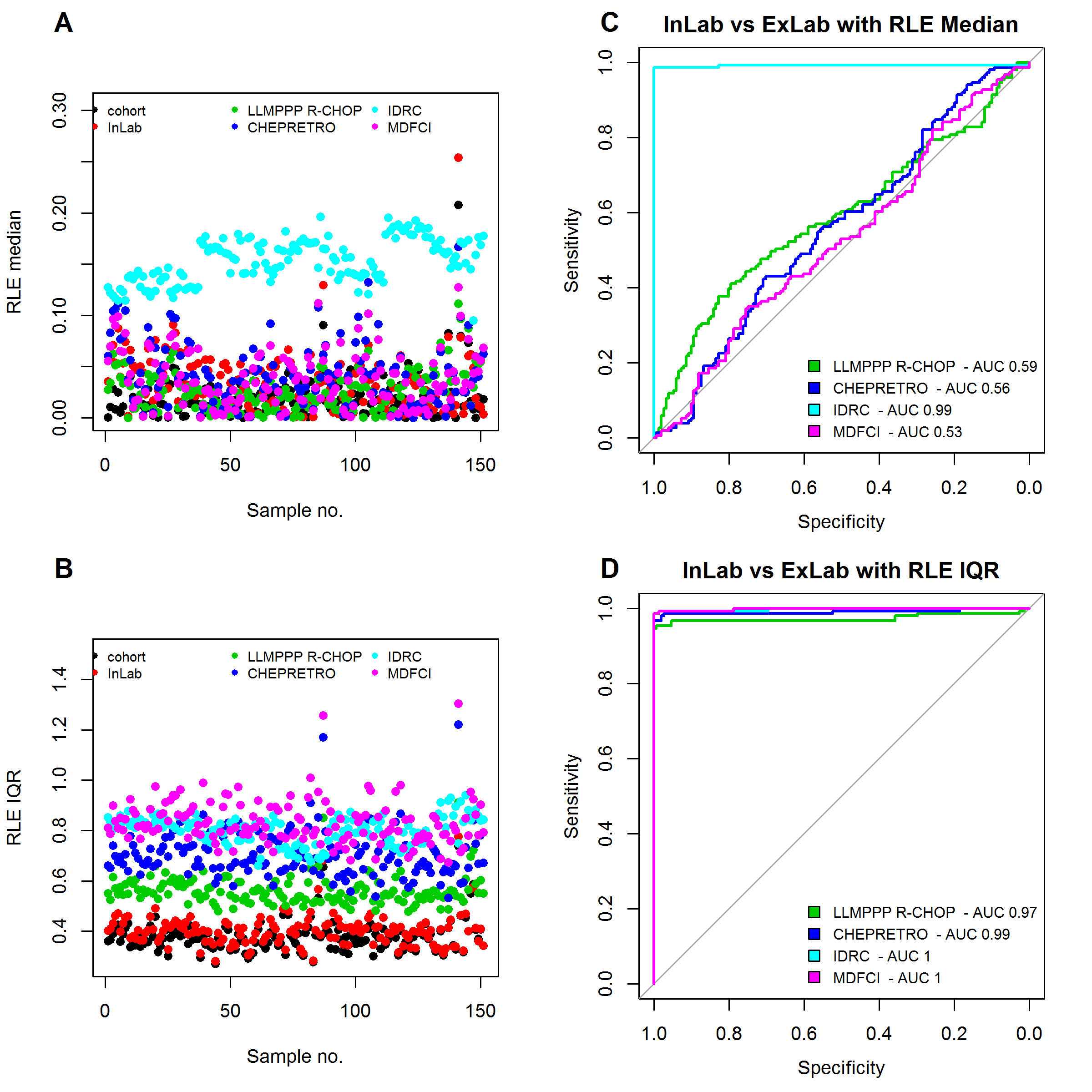

Supplement: S4 Fig — (TIFF) [file pone.0163711.s011.tiff]

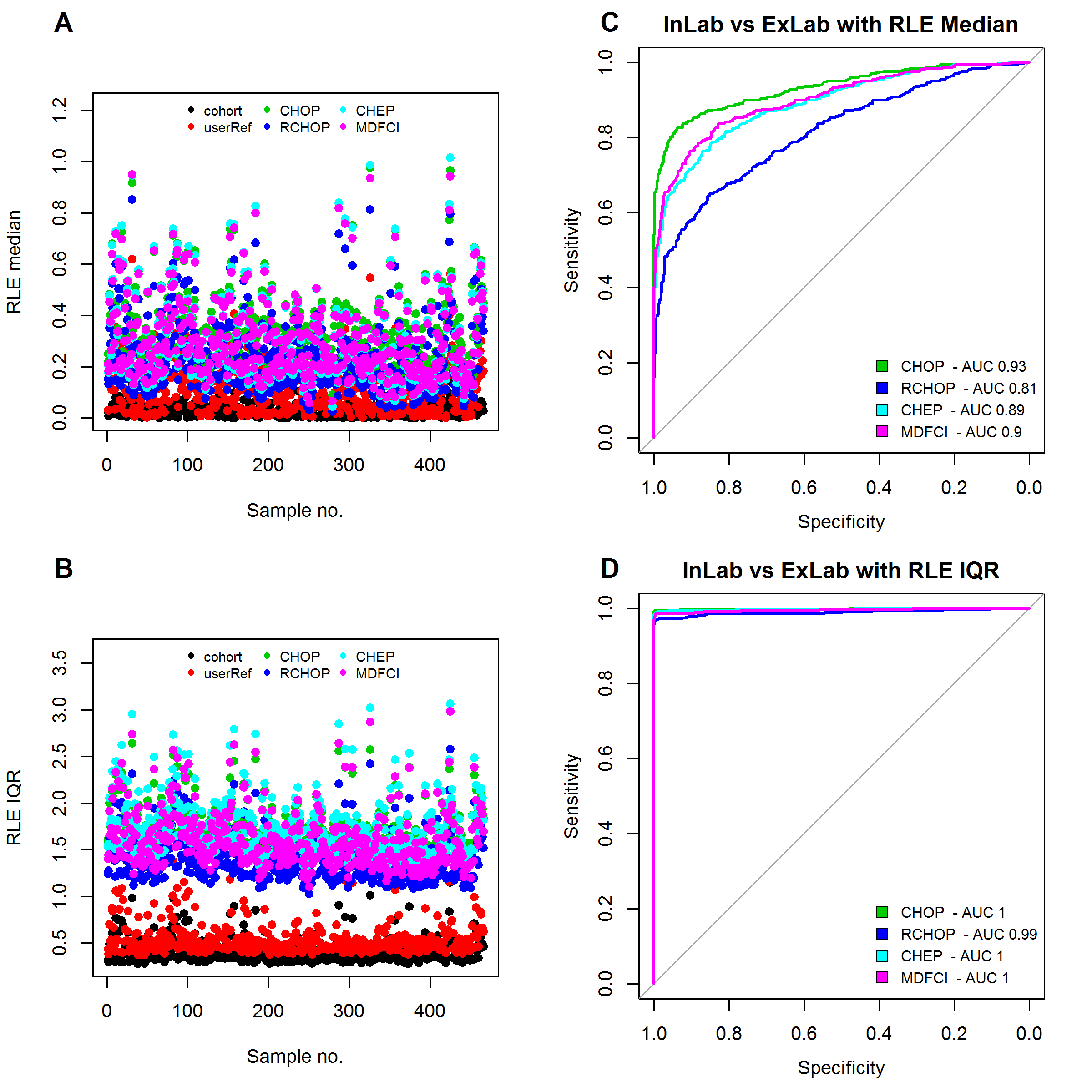

Supplement: S5 Fig — (TIFF) [file pone.0163711.s012.tiff]

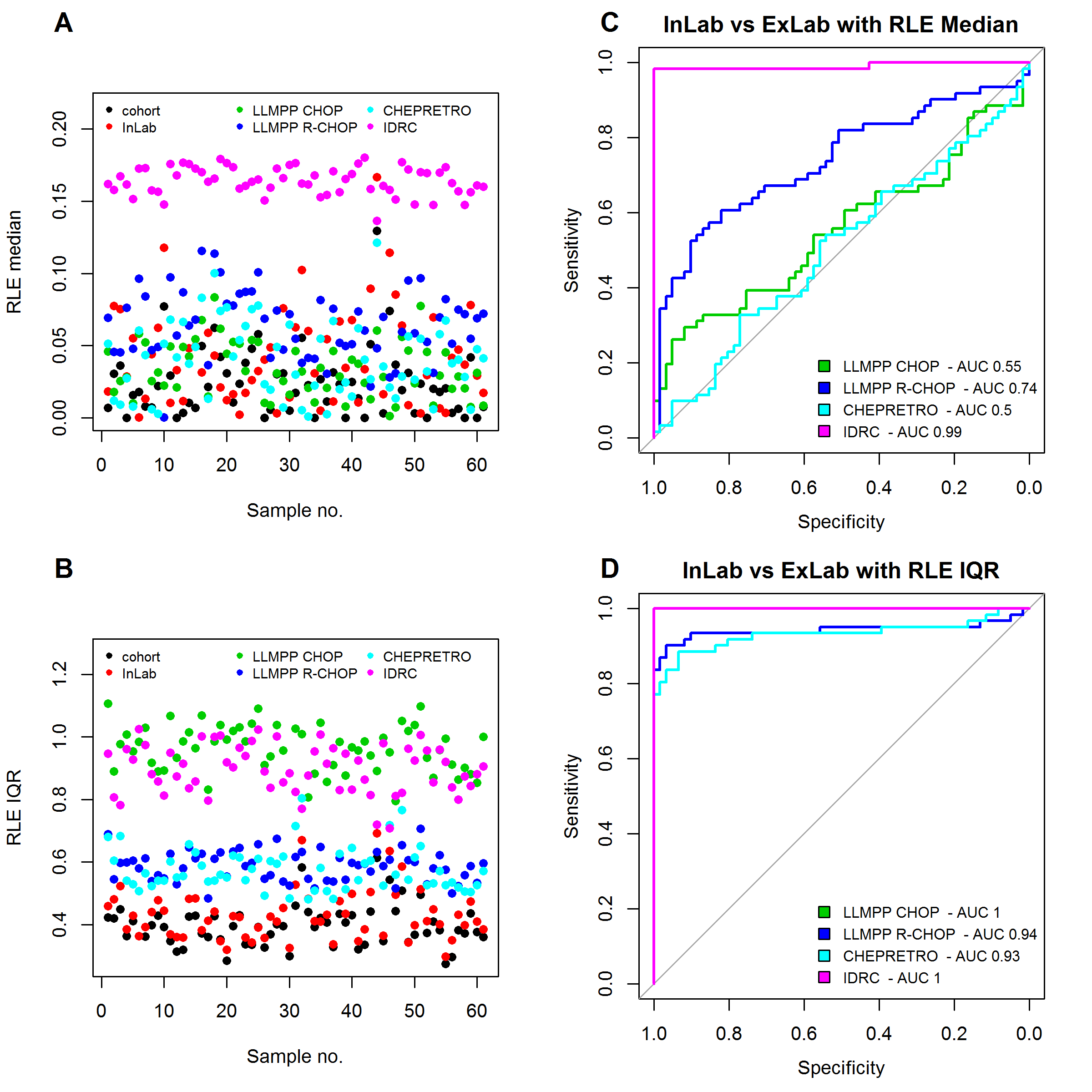

Supplement: S6 Fig — (TIFF) [file pone.0163711.s013.tiff]

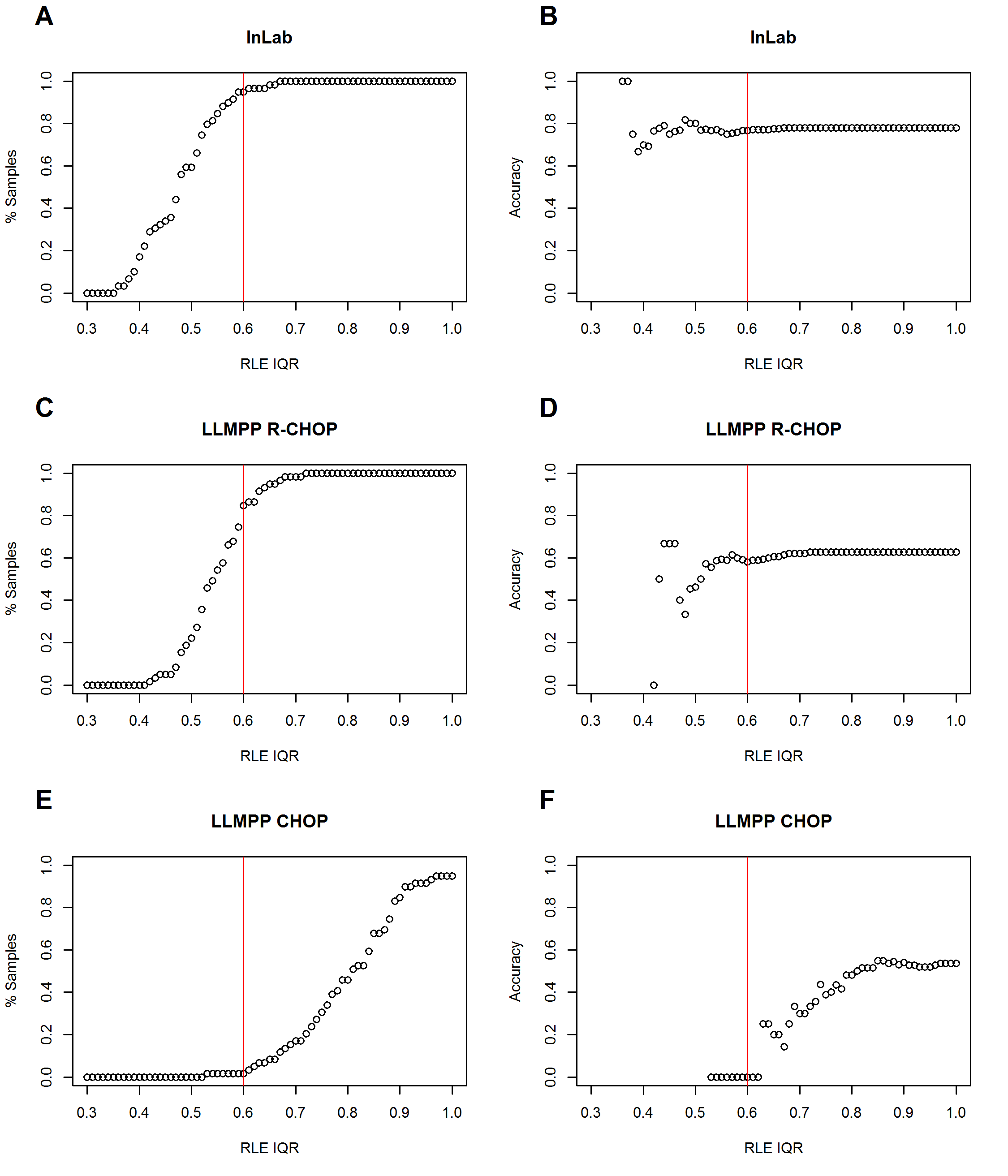

Supplement: S7 Fig — (TIFF) [file pone.0163711.s014.tiff]

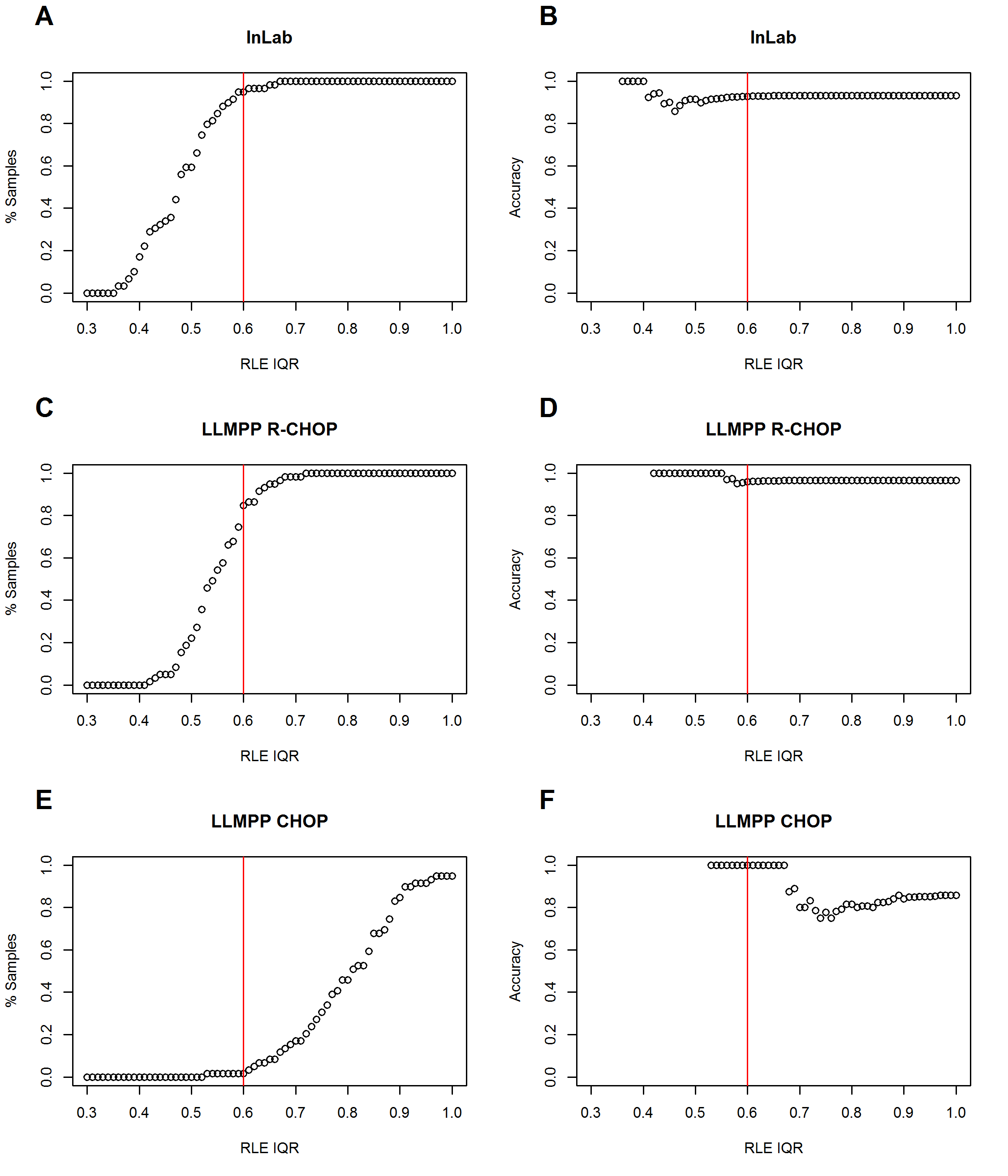

Supplement: S8 Fig — (TIFF) [file pone.0163711.s015.tiff]

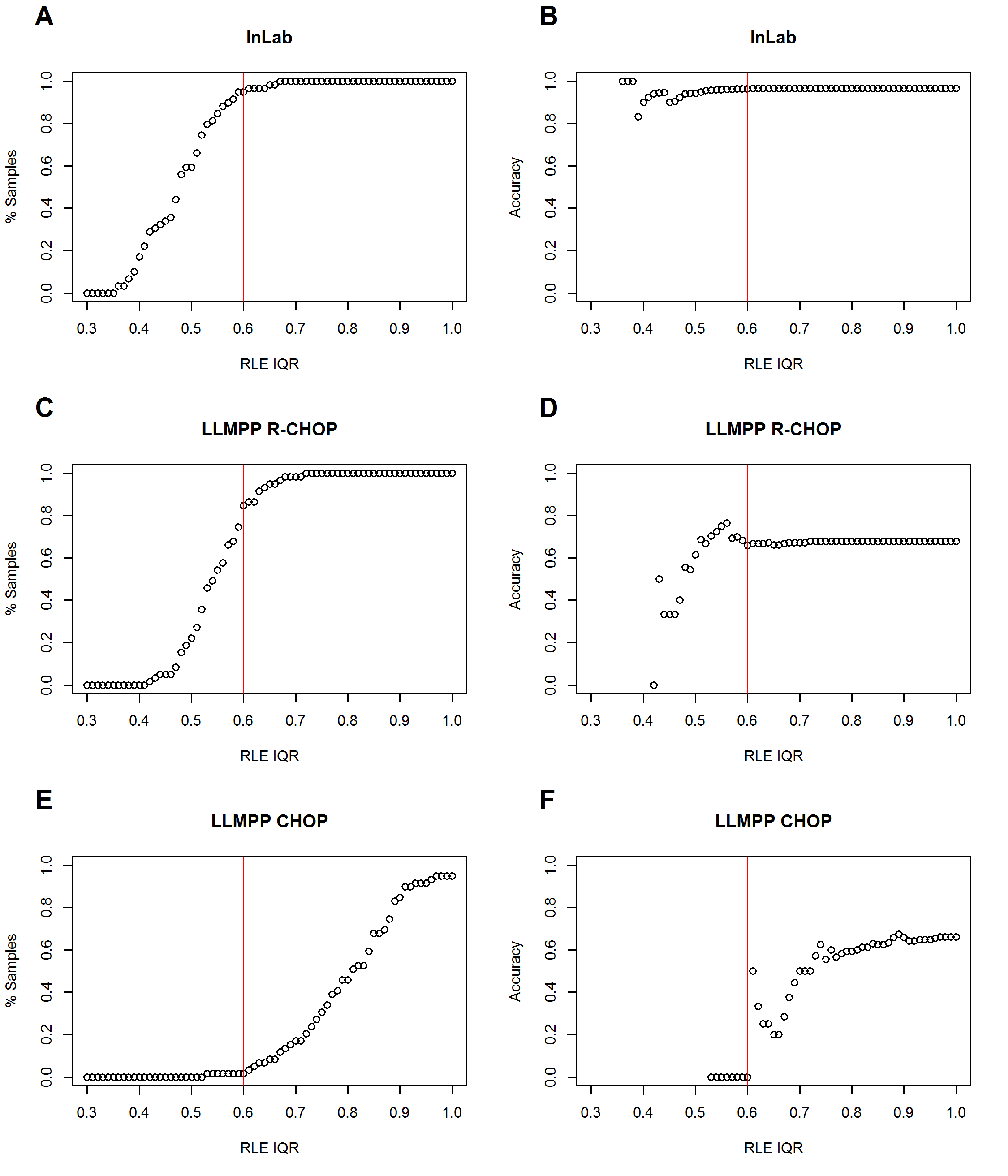

Supplement: S9 Fig — (TIFF) [file pone.0163711.s016.tiff]

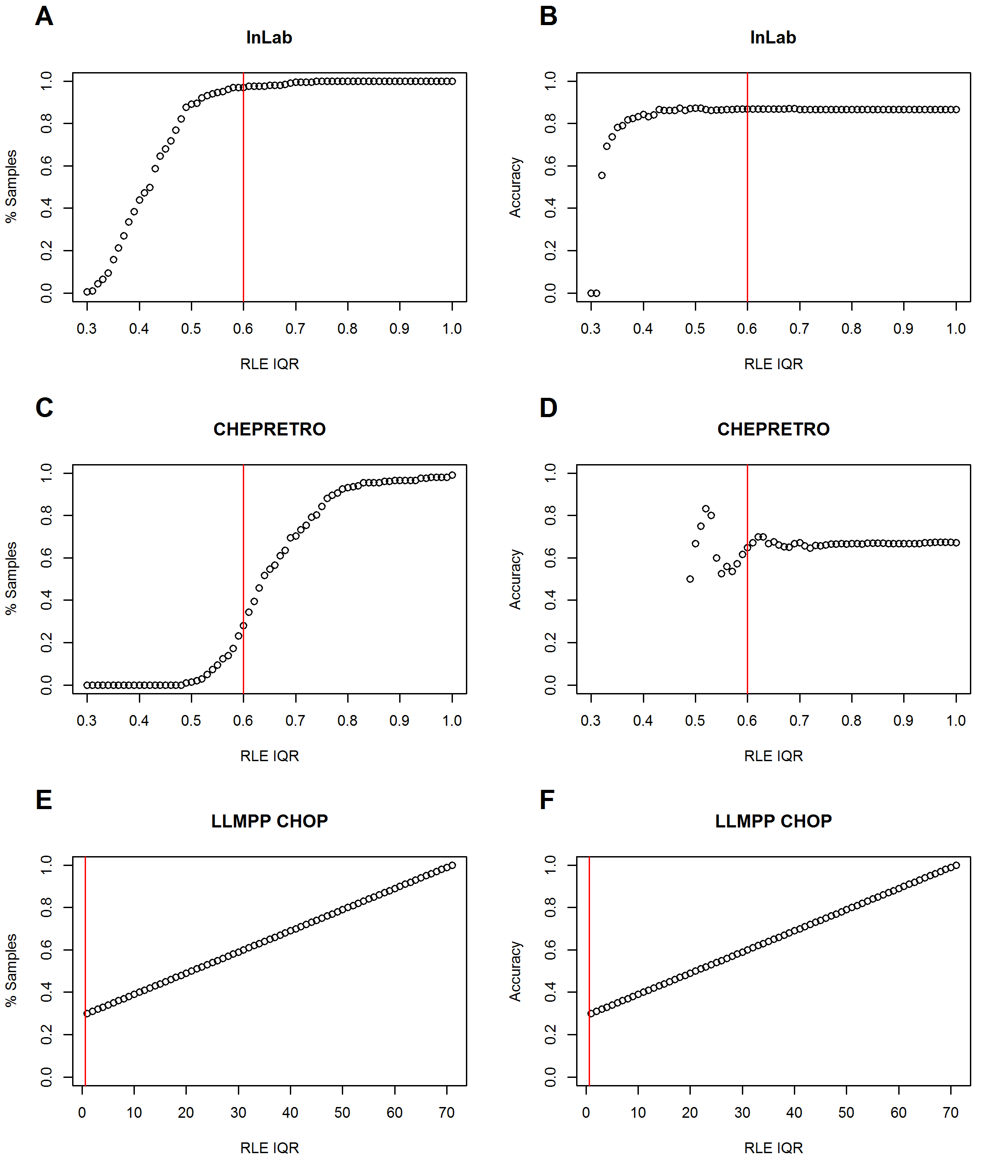

Supplement: S10 Fig — (TIFF) [file pone.0163711.s017.tiff]

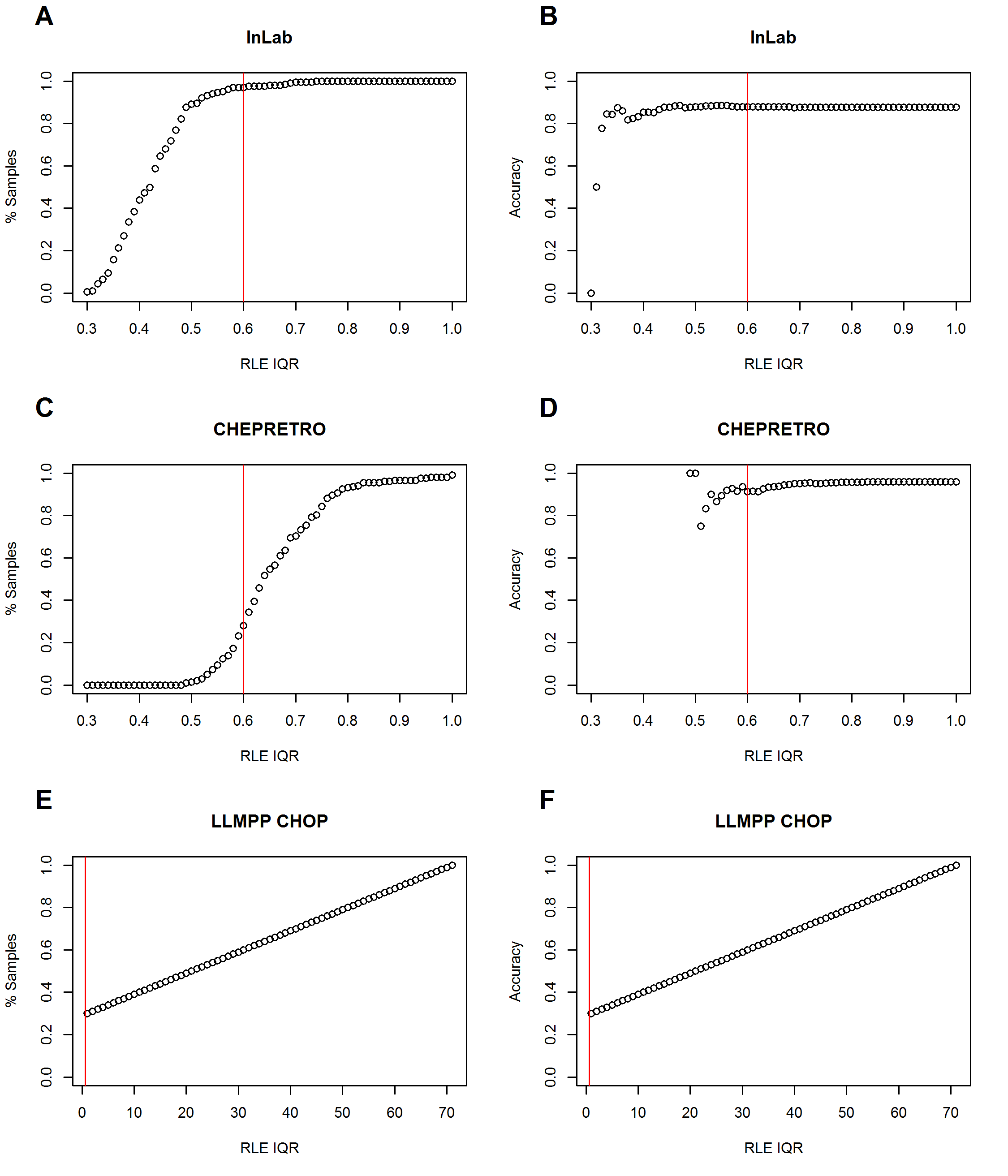

Supplement: S11 Fig — (TIFF) [file pone.0163711.s018.tiff]

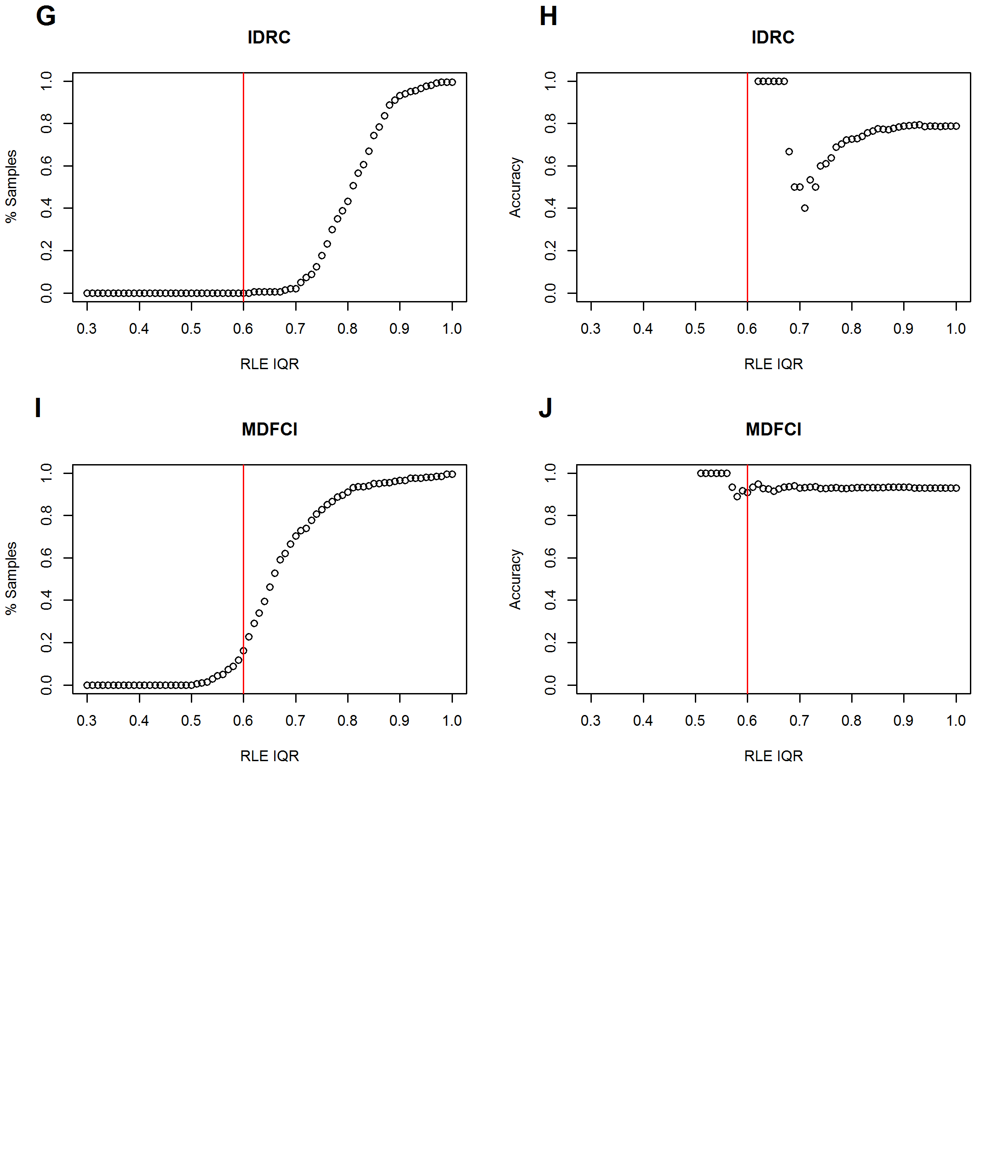

Supplement: S12 Fig — (TIFF) [file pone.0163711.s019.tiff]
